# Can PSMA-Targeting Radiopharmaceuticals Be Useful for Detecting Hepatocellular Carcinoma Using Positron Emission Tomography? An Updated Systematic Review and Meta-Analysis

**DOI:** 10.3390/ph15111368

**Published:** 2022-11-08

**Authors:** Alessio Rizzo, Manuela Racca, Domenico Albano, Francesco Dondi, Francesco Bertagna, Salvatore Annunziata, Giorgio Treglia

**Affiliations:** 1 Department of Nuclear Medicine, Candiolo Cancer Institute, FPO—IRCCS, 10060 Turin, Italy; 2 Division of Nuclear Medicine, Università Degli Studi di Brescia and ASST Spedali Civili di Brescia, 25123 Brescia, Italy; 3 Unità di Medicina Nucleare, TracerGLab, Dipartimento di Diagnostica per Immagini, Radioterapia Oncologica ed Ematologia, Fondazione Policlinico Universitario A. Gemelli, IRCCS, 00168 Rome, Italy; 4 Clinic of Nuclear Medicine, Imaging Institute of Southern Switzerland, Ente Ospedaliero Cantonale, 6501 Bellinzona, Switzerland; 5 Faculty of Biology and Medicine, University of Lausanne, 1011 Lausanne, Switzerland; 6 Faculty of Biomedical Sciences, Università Della Svizzera Italiana, 6900 Lugano, Switzerland

**Keywords:** PSMA, PET, nuclear medicine, liver, hepatocellular carcinoma, oncology, imaging

## Abstract

Background: Several studies proposed the use of positron emission tomography (PET) with Prostate-Specific Membrane Antigen (PSMA)-targeting radiopharmaceuticals in hepatocellular carcinoma (HCC). Our aim is to calculate the detection rate (DR) of this examination in HCC with a meta-analysis. Methods: A comprehensive literature search of studies on the DR of PET/CT or PET/MRI with PSMA-targeting radiopharmaceuticals in HCC was performed. Original articles evaluating these imaging examinations both in newly diagnosed HCC patients and HCC patients with disease relapse were included. Pooled DR including 95% confidence intervals (95% CI) was calculated. Statistical heterogeneity was also assessed using the I^2^ test. Results: The meta-analysis of six selected studies (126 patients) provided a DR of 85.9% for PET imaging with PSMA-targeting radiopharmaceuticals in the diagnosis of HCC. Moderate statistical heterogeneity among the included studies was found (I^2^ = 56%). Conclusions: The quantitative data provided demonstrate the high DR of PET/CT or PET/MRI with PSMA-targeting radiopharmaceuticals for HCC lesion detection. However, more studies are needed to confirm the promising role of PSMA-targeted PET in HCC.

## 1. Introduction

Liver cancer is the sixth most common cancer worldwide and the fourth most prevalent cause of cancer-related death globally, and hepatocellular carcinoma (HCC) is the most common type of liver cancer, accounting for about 90% of cases [1,2]. The highest incidence and mortality of HCC are observed in East Asia and Africa, although HCC incidence and mortality are increasing in Europe and USA [2]. Moreover, Surveillance Epidemiology End Results (SEER) reported HCC being the fastest increasing cause of cancer-related death in the USA since the early 2000s. Should this trend continue, HCC will become the third leading cause of cancer-related death by 2030 [3]. Over 90% of HCC cases occur in the setting of chronic liver disease, and cirrhosis from any etiology is reported as the leading risk factor for HCC; other major risk factors for HCC include chronic alcohol consumption, diabetes, or obesity-related non-alcoholic steatohepatitis, and infection by HBV or HCV, whereas less prevalent risk factors for HCC are hemochromatosis and α1-antitrypsin deficiency [4,5]. The molecular pathogenesis of HCC varies according to the distinct genotoxic insults and etiologies [6].

Imaging is essential to HCC diagnosis and management, contributing to primary liver tumor typing and HCC staging [7]. To date, imaging in HCC mainly relies on computed tomography (CT) and magnetic resonance imaging (MRI) [8]. The latter demonstrated slightly superior performances compared to CT, particularly in small lesions, with specificity ranging from 85% to 100% [9]. Many authors compared the diagnostic performances of multiphasic CT with gadoxetic acid-enhanced MRI and observed that gadoxetic acid-enhanced MRI has higher sensitivity than multiphasic CT and similar specificity [10]. Moreover, several meta-analyses explored the performances of MRI using extracellular or hepatobiliary contrast agents and reported that hepatobiliary contrast agents are associated with higher sensitivity than extracellular agents. Despite its lower specificity for HCC diagnosis, gadoxetic acid-enhanced MRI has advantages over extracellular contrast agents since it has a higher sensitivity for detecting nodules that do not display the typical features of imaging hallmarks or high-grade dysplastic nodules [11]. Contrast-enhanced ultrasound (CEUS) is a relatively novel non-invasive instrument for HCC diagnosis that underwent drastic improvement in recent years since a retrospective large-cohort study reported that arterial phase hyperenhancement followed by late washout (>60 s) had a positive predictive value for HCC in almost 99% of cases with no misdiagnoses reported [12]. However, CEUS accounts for several limitations since it is not a panoramic technique, it can be employed to study one or very few nodules visible at conventional baseline US, and it suffers from the difficulty of reviewing images acquired in another center, unlike CT or MRI [13].

Molecular imaging has the advantage of detecting functional abnormalities that usually precede anatomical changes at morphological imaging in oncological diseases [14]. Concerning molecular imaging, the most studied examinations in HCC patients are Fluorine-18 fluorodeoxyglucose ([^18^F]FDG) and radiolabeled choline (with ^18^F or ^11^C) positron emission tomography/computed tomography (PET/CT) [15,16]. [^18^F]FDG PET/CT showed no valuable additional information for the early diagnosis of HCC because of its low diagnostic accuracy (lower than 40%), especially in well-differentiated HCCs [17]; nevertheless, it seems that [^18^F]FDG PET might have an added value in the detection of extrahepatic metastatic disease in advanced tumors but, considering the high diagnostic accuracy of CT and MRI imaging, its employment in clinical practice is poor in this setting [4]. About PET with radiolabeled choline, it showed a potential role in characterizing HCC lesions greater than 1 cm and detecting extrahepatic metastases; however, as for [^18^F]FDG PET, its use is limited by the overwhelming CT and MRI performances [18].

Prostate-specific membrane antigen (PSMA), also known as glutamate carboxypeptidase type II, is a transmembrane protein encoded by the gene FOLH1, firstly discovered in prostate cancer cells in 1987 [19,20]. In the following years, it was observed that PSMA is not exclusively expressed by prostate cancer cells but is also expressed on the surface of other types of cancer cells and neovascular endothelial cells of various solid tumors [21]. In this setting, several low-molecular-weight radiolabeled PSMA inhibitors have been developed to improve diagnostic performances of nuclear medicine imaging and as theragnostic agents for prostate cancer patients, demonstrating an effective diagnostic compound and therapeutic agent [22,23]. Since PSMA is overexpressed by neovascular endothelium cells of various malignancies, including HCC, this could be a rationale to employ PET/CT or PET/MRI with PSMA-radioligands in tumors with low [^18^F]FDG uptake, evaluating other molecular pathways than the glucose metabolism. Moreover, since first-line therapy in locally advanced and metastatic HCC consists of a combination of immunotherapy and anti-neoangiogenic treatment [1], PET/CT with PSMA-radioligands might be a valuable instrument to predict the outcome of the therapy and assess the response to the ongoing treatment.

Several recent studies employed PET imaging with PSMA-radioligands for HCC. This paper aims to perform a systematic review and meta-analysis to calculate the detection rate (DR) of PET with PSMA-targeting radioligands in patients with HCC. As a secondary objective, this article aims to collect evidence about the comparison of diagnostic performance among PET with PSMA-radioligands and other imaging methods in HCC.

## 2. Materials and Methods

### 2.1. Protocol

The present systematic review and meta-analysis were conducted following a predefined protocol [24], and the “Preferred Reporting Items for a Systematic Review and Meta-Analysis” (PRISMA 2020 statement) were used as a benchmark in its production [25]. The complete PRISMA checklist is available as Supplementary Material (Table S1). No pre-registration was accomplished.

As the first step, a straightforward review question was defined as follows: can PSMA-targeting radiopharmaceuticals be useful for detecting HCC using PET imaging?

A literature search according to the Population, Intervention, Comparator, Outcomes (PICO) framework was performed, establishing the criteria for study eligibility as follows: patients with HCC diagnosis (Population), undergoing PSMA-targeted PET (Intervention) compared or not with conventional imaging (Comparator); the considered outcomes were the evaluation of PSMA radioligand uptake in HCC and PSMA-targeted PET DR in HCC.

Two reviewers (one expert on the topic and one non-expert to avoid selection bias) independently performed the literature search, the selection of the studies, and the quality assessment. An online consensus meeting solved any discrepancies among the reviewers.

### 2.2. Literature Search Strategy and Information Sources

Defined the review question, two authors (A.R. and G.T) independently carried out a comprehensive literature search employing two electronic bibliographic databases (PubMed/MEDLINE and Cochrane library), searching for papers assessing the diagnostic accuracy of PSMA-targeting PET in HCC. The database ClinicalTrials.gov was also used to search for ongoing studies (access date: 22 August 2022).

A search algorithm based on a combination of these terms was used: (A) “PSMA” AND (B) “hepatocellular” OR “liver” OR “hepatic” OR “HCC”. No restrictions were applied regarding the year of publication or article language. Furthermore, references to included studies were also screened to find additional eligible articles to refine the research. The literature search was lastly updated on 22 August 2022.

### 2.3. Eligibility Criteria

The authors considered as eligible for inclusion in this systematic review and meta-analysis clinical studies reporting information on the employment of PSMA radioligand PET in the staging and restaging of HCC. Exclusion criteria for the systematic review (qualitative analysis) were predefined: reviews, letters, comments, editorials on the topic of interest, case reports or small case series on the analyzed subject, and original articles dealing with different fields of interest (including preclinical studies or studies not using PSMA-radioligand PET in HCC) were excluded. Exclusion criteria for the meta-analysis (quantitative analysis) were also predefined: articles not providing sufficient information to reassess the DR of PSMA radioligand PET and studies with possible patient data overlap with another paper were excluded.

### 2.4. Selection Process

Based on the predefined inclusion/exclusion criteria and the literature search strategy, two authors (A.R and G.T.) independently reviewed titles and abstracts of the obtained papers. The assessment for the final inclusion of the selected studies was independently carried out for the systematic review and the meta-analysis. The reviewers decided about the exclusion or inclusion in the review specifying the reason for all the screened records using the bibliographic databases.

### 2.5. Data Collection Process and Data Extraction

Two authors (A.R. and G.T.) independently collected all the included studies to avoid possible biases and extracted data taking advantage of the information in full text, tables, and figures. The following data were extracted for every single study included in the systematic review: patient characteristics (sample size, age, sex ratio, clinical setting, and other diagnostic imaging); general study information (authors, country, publication year, study design, funding sources); patient information (sample size, age, sex ratio, clinical setting, and other diagnostic imaging); index text characteristics (employed PSMA-radioligand, type of hybrid imaging protocol, patient preparation, radiopharmaceutical administered activity, uptake time between PSMA-radioligand administration and image acquisition, and the protocol for image analysis); data on the DR of PSMA-radioligand PET in HCC on a per-patient-based analysis; and the diagnostic benchmark used.

### 2.6. Quality Assessment (Risk of Bias Assessment)

QUADAS-2, a tool for evaluating quality in diagnostic test accuracy studies, was used for assessing the risk of bias in individual studies and the applicability to the review question [26]. Two reviewers (A.R and G.T) independently assessed the quality of the studies included in the systematic review and meta-analysis. Four domains (patient selection, index test, reference standard, and flow and timing) were assessed regarding the risk of bias, and three fields were evaluated regarding applicability (patient selection, index test, and reference standard). Any disagreement between the authors about the quality assessment were solved by an online consensus meeting.

### 2.7. Effect Measures

The primary outcome of the meta-analysis was the DR of PSMA-targeted PET in HCC. DR was defined as the ratio between the number of scans with at least one suspected HCC lesion detected and the total number of scans performed.

Other secondary outcome measures were described in the qualitative synthesis (systematic review), taking into account the information provided in the results section of the included studies.

### 2.8. Statistical Analysis

The authors performed a pooled analysis of the DR of PSMA-targeted PET, employing data from the included studies and taking into account the weight of each study using a random-effects statistical model as suggested by DerSimonian and Laird [24]. Furthermore, 95% confidence interval values (95% CI) were provided and subsequently displayed employing forest plots.

The I-square index (I^2^) or inconsistency index was used to estimate the statistical heterogeneity among the included studies; a significant statistical heterogeneity was defined whether I^2^ was >50% [24,27].

Moreover, publication bias was assessed through a visual analysis of the symmetry/asymmetry of the funnel plot or by using Egger’s test whether less than six studies were included in the meta-analysis [24].

Statistical analyses were performed using MedCalc statistical software version 18.2.1 (MedCalc Software bvba, Ostend, Belgium).

### 2.9. Additional Analyses

In case of significant statistical heterogeneity of the included studies, subgroup analyses were taken into account based on study design, patient characteristics, technical aspects, and explored clinical settings.

## 3. Results

### 3.1. Literature Search and Study Selection

**Figure 1:**
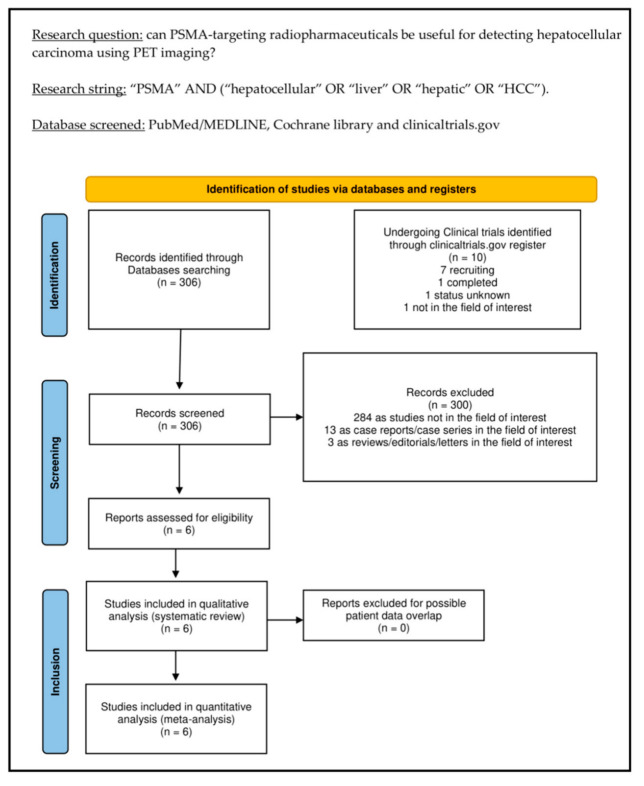
Summary of the study selection process for the systematic review and meta-analysis.

The comprehensive literature search (last update: 22 August 2022) revealed 306 records. Based on the predefined inclusion and exclusion criteria, these 306 papers were assessed for eligibility, and 300 papers were excluded (13 as reviews, letters, or editorials; 3 as case reports; and 284 as not in the field of interest). Six records were appropriate for inclusion in the systematic review (qualitative synthesis) and meta-analysis (quantitative synthesis) after full-text assessment [28,29,30,31,32,33]. The screening of the references of these papers did not provide any additional eligible study. Figure 1 summarizes the study selection process.

### 3.2. Study Characteristics

The comprehensive characteristics analysis of the six papers eligible for the systematic review (qualitative analysis), including 126 HCC patients, are presented in Table 1, Table 2 and Table 3. Concerning general study information (Table 1), the included studies were published between 2018 and 2022 in Europe, Asia, and USA. All studies but one had a prospective design; each was conducted in a single center, and none of the revised articles declared funding in their text.

Concerning the patient key characteristics (Table 2), the sample size ranged from 7 to 40 HCC patients (mean/median age range: 56–66 years; male percentages ranged from 71% to 93%). The index test was employed for staging in one study [33], to restage the disease in one study [29], and for both purposes in four studies [28,30,31,32]. Three studies evaluated the Child-Pugh score of the included patients; among them, the most represented was the A score (50 patients) [28,30,32]. Comparative imaging examination was only [^18^F]FDG PET/CT in one study [29], only contrast-enhanced CT (ceCT) in one study [32], [^18^F]FDG PET/CT with additional MRI in one study [31], [^18^F]FDG PET/CT with additional ceCT and MRI in one study [28], and ceCT with additional MRI in the remaining two studies [30,33].

Several differences among the included studies were found in the index test key characteristics (Table 3). In all studies, the administered radiopharmaceutical was [^68^Ga]Ga-PSMA-11, and the injected activity ranged from 148 to 185 MBq (in absolute values) and between 2 and 2.5 MBq/Kg (in relative values). In contrast, the time interval between PSMA-targeting radiopharmaceutical injection and PET imaging acquisition ranged from 60 to 90 min.

All the included studies co-registered PET imaging with CT, although in one study, five of the included patients underwent PET/MRI instead of PET/CT [33].

PET imaging interpretation was performed both with qualitative and semi-quantitative analyses in all the included studies. Semi-quantitative analyses included the calculation of the maximal, minimal, and mean standardized uptake values (SUV_max_, SUV_min_, and SUV_mean_). Target-to-background uptake ratio (TBR) was calculated in all the included studies and was obtained by dividing lesions’ SUV_max_ for the healthy liver SUV_mean_.

### 3.3. Risk of Bias and Applicability

**Figure 2:**
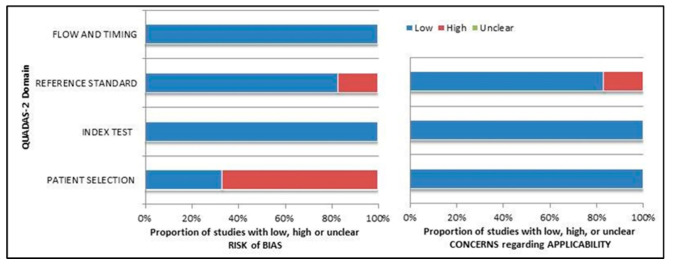
Summary of quality assessment according to QUADAS-2 tool. Studies included in the systematic review are classified as low risk or high risk of bias or applicability concerns for different domains (reported in the vertical axis). The horizontal axis indicates the percentage of studies. The graph suggests that more than 60% of the studies show a potentially high risk of bias in patient selection.

The comprehensive evaluation of the risk of bias and concerns about the applicability of studies included in the systematic review according to QUADAS-2 is presented in Figure 2.

### 3.4. Results of Individual Studies (Qualitative Synthesis)

Overall, PSMA-targeting PET/CT or PET/MRI showed excellent diagnostic performance in detecting HCC lesions in all the studies included in the systematic review, both on a per-patient- and per-lesion-based analyses and in different clinical settings. Moreover, PET/CT or PET/MRI with PSMA-targeting radiopharmaceutical detected extrahepatic lesions in lymph nodes, peritoneum, and bone [28,29,30,31,32,33].

PSMA-targeting radiopharmaceuticals safety profile was evaluated only in one study [30], which did not report any adverse event after the administration of [^68^Ga]Ga-PSMA-11. When reported, the image quality of PSMA-targeting PET images was classified as excellent and interobserver agreement was moderate [32].

All the included studies reported variable uptake of PSMA-targeting radiopharmaceuticals in HCC lesions; in most cases, it was higher than the uptake of the surrounding liver parenchyma [28,29,30,31,32,33]. Concerning semiquantitative metrics, average SUV_max_ reported values ranged between 8.3 and 16.7, while average TBR values ranged from 2 to 3.6 [28,29,30,31,32,33]. No significant differences in SUV or TBR between the explored clinical settings (staging and restaging) were found [30]. Moreover, one study focalized on PSMA-targeting radiopharmaceuticals in differentiating HCC lesions from regenerative nodules in cirrhotic HCC patients, revealing non-significant uptake in the latter [28].

Compared to [^18^F]FDG as PET/CT radiopharmaceutical, PSMA-targeting agents showed a greater number of positive patients and lesions; furthermore, when [^18^F]FDG and PSMA uptake were compared in positive lesions, PSMA PET/CT showed higher values of SUV_max_ and TBR [28,29,31]. Compared to CT and MRI, PSMA-targeting PET/CT showed slightly superior performance in detecting hepatic and extrahepatic HCC lesions [28,30,31,32]. Interestingly, the radiopharmaceutical uptake was significantly higher in contrast-enhancing tumor areas than in non-enhancing regions [28].

Three of the included studies correlated the in vivo and in vitro PSMA expression in HCC using PSMA-targeted PET and immunohistochemistry staining for PSMA, respectively [28,30,33]. In this context, intense PSMA staining was reported in the neovascular endothelium of lesions that showed high uptake of PSMA-targeting radiopharmaceuticals in PET images, whereas PSMA staining was absent in the cell membrane and cytoplasm of HCC cells.

Finally, two studies correlated PET semi-quantitative parameters (SUV_max_) with laboratory exam results. No statistically significant relationships were found with the serum concentrations of alpha-fetoprotein, CA 19-9, and CEA [30,31].

### 3.5. Meta-Analysis (Quantitative Synthesis)

Six studies, including 126 HCC patients, were selected for the pooled analysis of the DR of PSMA-targeted PET. Overall, the DR of PSMA-targeted PET for detecting HCC ranged from 67.7% to 100% (Table 4).

**Figure 3:**
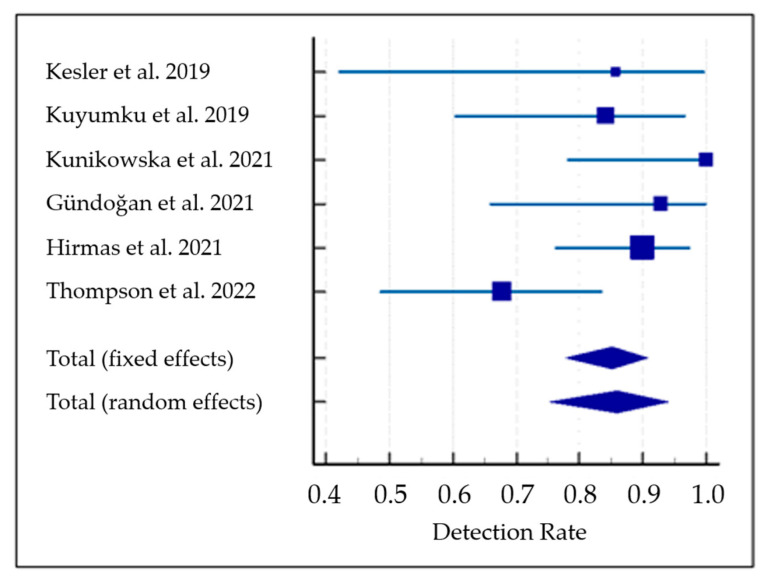
Meta-analysis on the detection rate of PET with PSMA-targeting radioligands in patients with hepatocellular carcinoma [28,29,30,31,32,33]. Blue lines indicate the 95% confidence interval values and blue squares indicate the detection rate value of each study. Trapezoid indicates the pooled value with 95% confidence interval values.

**Figure 4:**
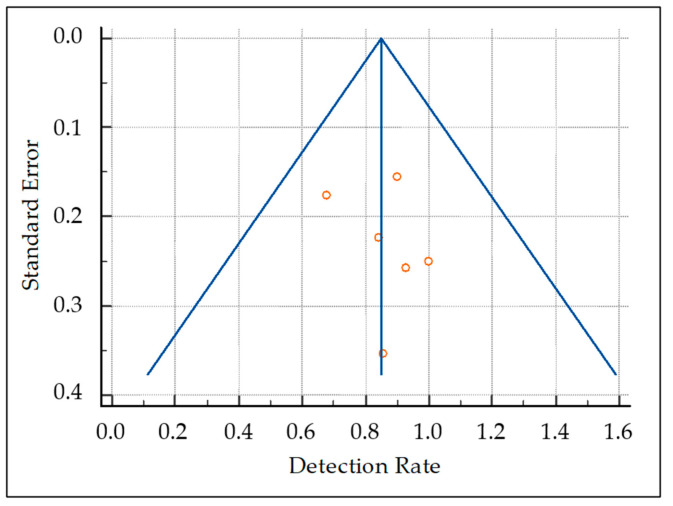
Funnel plot for publication bias assessment. The presence/absence of publication bias is judged taking into account the asymmetric/symmetric distribution of studies (red circles) in the funnel plot.

The pooled DR was 85.1% (95% confidence interval (95% CI): 77.9–90.7%) (Figure 3). A moderate statistical heterogeneity among the included studies was found as I^2^ was 56%. The funnel plot for publication bias assessment is illustrated in Figure 4. There is no significant asymmetry in the funnel plot, supporting the absence of substantial publication bias.

Considering the statistical heterogeneity observed, a subgroup analysis omitting the only study that employed PET/MRI as hybrid imaging modality [33] was performed. The subgroup analysis showed a pooled DR of 89.6% for PSMA-targeted PET/CT (95% CI: 83–94.8%) without statistical heterogeneity among the included studies (I^2^: 0%).

## 4. Discussion

On account of its overexpression on the cell membrane of prostate cancer cells, PSMA is an effective target for molecular imaging and radioligand therapy (RLT) in the management of prostate cancer patients. A growing body of literature reporting outstanding results is emerging, expanding their application in different clinical settings [34,35]. Nevertheless, PSMA is not selectively expressed in prostate cancer cells, but its presence is also reported in neo-vascular endothelial cells of different cancers [36,37]. Furthermore, preclinical studies postulated that PSMA might regulate tumor cell invasion and neo-angiogenesis by modulating integrin signal transduction in endothelial cells [38]. In this context, the presence of PSMA expression in HCC neo-vasculature is the rationale for employing PSMA-targeting radiopharmaceuticals for imaging and therapy in these malignancies [21].

In the past four years, several papers assessed the diagnostic performance of PET imaging with PSMA-targeting radiopharmaceuticals to detect HCC lesions in newly diagnosed patients as well as in patients previously submitted to various lines of local or systemic therapy [28,29,30,31,32,33]. In this meta-analysis, we pooled published data to increase the statistical power and, subsequently, to accomplish more robust detection rate estimates than the original studies.

Even though the available literature data are quite limited, an excellent DR of PET/CT or PET/MRI with PSMA-targeting radiopharmaceuticals in HCC patients has been reported both in patients undergoing initial staging of their disease and restaging for recurrence, without any statistical difference in performance between these clinical settings [30].

Overall, PET imaging with PSMA-targeting radiopharmaceuticals demonstrated slightly superior performance compared to CT and MRI for detecting hepatic and extrahepatic HCC lesions [28,30,31,32]; moreover, it was observed that radiopharmaceutical uptake was significantly higher in contrast-enhancing tumor areas than in non-enhancing ones [28]. Since contrast enhancement in CT imaging is closely correlated to micro-vessel density [39], this finding is consistent with the tumor neo-vasculature staining for PSMA, and the subsequent uptake highlighted in PET images. Furthermore, no significant uptake of PSMA-targeting radiopharmaceuticals was reported in regenerative nodules in cirrhotic patients [28]. This finding is in line with a recent article published by Chen et al., who reported the absence of PSMA staining in most of the cirrhotic liver specimens they analyzed (28/30) and weak PSMA staining in the remaining (2/30) [40]. Since the cirrhotic livers have a nodular architecture with altered vascularity, which makes challenging the differentiation between regenerative nodules and HCC lesions in traditional imaging methods, this potential employment of PSMA-targeting radiopharmaceuticals needs to be further explored.

Overall, contrast-enhanced CT and MRI remain the gold-standard imaging methods for evaluating HCC [4]. However, PET with PSMA-targeting radiopharmaceuticals could be a complementary examination when conventional imaging is doubtful. Further studies should assess the actual diagnostic advantage of PSMA-targeted PET over contrast-enhanced CT and MRI in HCC. Moreover, considering the biological mechanism underlying PSMA-targeting radiopharmaceuticals uptake in HCC, PSMA-targeted PET/CT has excellent potential in the prediction and assessment of treatment response, but more prospective studies comparing its performances with CT and MRI in these settings are needed to evaluate its exact role as well as the correct timing of the functional imaging evaluation based on the type of treatment the patients were submitted to.

[^18^F]FDG PET/CT has limitations for staging and restaging of HCC [17]. [^18^F]FDG uptake is usually observed in metabolically active cells; thus, cancer cells should show higher avidity for this radiopharmaceutical than normal cells. In most normal and tumoral cells, [^18^F]-FDG is phosphorylated by hexokinase to [^18^F]FDG-6-phosphate, which cannot be metabolized and remains within the cell as a polar metabolite that can be visualized by PET imaging; nevertheless, in liver cells, [^18^F]FDG-6-phosphate can undergo dephosphorylation and can exit the cells. Since the enzymes of well-differentiated HCC are similar to the ones observed in normal liver, this could explain the mild [^18^F]FDG uptake expressed by well-differentiated HCC and the subsequent low sensitivity of [^18^F]FDG PET imaging [17]. Moderately to poorly differentiated HCC have lower levels of glucose-6-phosphatase and higher levels of hexokinase, justifying the higher [^18^F]FDG uptake compared to well-differentiated HCC [41,42]. Consistently with these data, recent reviews suggested that [^18^F]FDG PET/CT in HCC is a strong predictor of prognosis and has a role in the assessment of tumor size or number of tumor nodules (as defined by the Milan criteria) [43]. Based on the available literature [28,29,31], PET with PSMA-targeting radiopharmaceuticals showed higher diagnostic performances than [^18^F]FDG PET/CT. This finding may be explained by the different mechanisms underlying the uptake of the compared tracers as neo-angiogenesis is a rate-limiting factor in tumor growth [44], whereas [^18^F]FDG metabolism may be highly variable in HCC depending on its differentiation. In this context, it is fundamental to specify that not all kinds of intra-tumor neo-vascular endothelium has significant PSMA expression and that some kinds of tumors show weak neo-angiogenesis; this implies that PSMA-targeting radiopharmaceuticals might not be employed in every type of cancer [21].

No articles comparing PET with PSMA-targeting radiopharmaceuticals to [^11^C]acetate and choline-labeled (with [^18^F] or [^11^C]) radiopharmaceuticals were found in the literature search.

Acetate can be used by cells to synthesize cholesterol and fatty acids or oxidized via the tricarboxylic acid cycle to produce energy [45]. Thanks to its metabolism, [^11^C]acetate PET is a valuable instrument for studying tumors that rely more on fatty acid metabolism than glycolysis, such as low-grade HCC [46]. Therefore, [^11^C]acetate has the potential to play a complementary role to [^18^F]FDG PET/CT in the diagnostics of HCC [46]. Based on the available literature and the complementarity reported between [^18^F]FDG and [^11^C]acetate in PET imaging, it is possible that PSMA-targeting radiopharmaceuticals might demonstrate superior diagnostic performance than [^11^C]acetate PET imaging; nevertheless, since no studies compared the two examinations, it is not possible to express a definitive comparison between the examinations.

Choline-labeled tracers allow the study of lipid metabolism. Their use is based on the presence of large amounts of choline in HCC, as reported by proton magnetic resonance (MR) spectroscopy studies [47]. Choline is one of the components of phosphatidylcholine, an essential element of cell membrane phospholipids [48]. Malignant tumors may show a high proliferation and increased metabolism of cell membrane components, leading to increased uptake of choline [49]. Despite the fact that the current literature data do not provide studies that directly compare PSMA-targeting radiopharmaceuticals to choline-labeled tracers, our meta-analysis demonstrated that the DR of PET with PSMA-targeting radiopharmaceuticals for HCC is not inferior to the one observed in PET with choline-labeled radiopharmaceuticals, as observed in a published meta-analysis on radiolabeled choline PET which reported a DR of 84% in 115 HCC patients [15].

To date, an increasing amount of PSMA-targeting radiopharmaceuticals is available for nuclear medicine physicians, including novel [^18^F]-labelled compounds as [^18^F]F-DCFPyL and [^18^F]F-PSMA-1007 [50,51]. Based on the literature data available, the only PSMA-targeting radiopharmaceutical used for detecting HCC lesions was [^68^Ga]Ga-PSMA-11 [28,29,30,31,32,33]. Using [^18^F]-labeled PSMA-targeting radiopharmaceuticals seems to rely on several advantages since it can count on large-scale production, reduced costs, and high-quality images through lower positron energy and a longer half-life [52]. However, [^18^F]F-PSMA-1007 shows a different biodistribution from the other PSMA-targeting radiopharmaceuticals, demonstrating higher lipophilicity and, consequently, may not be found in the ureters and bladder since its clearance is liver-dominant [53]; this characteristic makes theoretically [^18^F]F-PSMA-1007 less suitable for HCC imaging than the others PSMA-targeting radiopharmaceuticals. In this context, more studies are needed to assess the actual feasibility of PSMA-radiopharmaceuticals other than [^68^Ga]Ga-PSMA-11 to be employed in the diagnostics of HCC.

An interesting argument underlying the employment of PSMA-targeting radiopharmaceuticals in HCC patients relies on the location of PSMA expression in intra-tumoral neo-vasculature. Considering that first-line therapy in unresectable and metastatic HCC is based on a synergic approach of antiangiogenic therapy and immunotherapy (bevacizumab/atezolizumab) [1], PSMA-targeting radiopharmaceuticals might play a role in the prediction and the assessment of treatment response, especially in cases where conventional imaging is doubtful. Since no studies deepened the potential role of PSMA-targeting PET in this clinical setting to date, prospective studies are warranted in order to overpower our diagnostic instruments as well as reach the goal of a truly tailored medicine in HCC patients.

One of the most exciting aspects of PSMA expression in HCC lesions relies on the concept of PSMA-targeted theragnostic, hence the use of PSMA both as a diagnostic and a therapeutic target. As reported by the recent VISION trial, PSMA-targeted therapy with [^177^Lu]Lu-PSMA-targeting radiopharmaceuticals provided excellent results in metastatic castration-resistant prostate cancer patients [22]. Based on this report, this treatment might also offer beneficial effects in other cancers characterized by PSMA overexpression in tumor cells or the neo-vasculature [54]. One of the studies included in this review performed [^177^Lu]Lu-PSMA-617 RLT in two patients who showed liver lesions with high uptake of [^68^Ga]Ga-PSMA-11 in PET images and had no other local or systemic treatment options [32]. In both cases, intra-therapeutic SPECT/CT-based dosimetry revealed that tumor radiation dose was at least 10-fold lower than typically accomplished by one cycle of external-beam radiation therapy for HCC, so this treatment modality was not as effective as anticipated and was suspended after one administration. Considering the low toxicity of PSMA-targeted RLT [55], further preclinical and clinical investigations are warranted to define a PSMA-targeted theragnostic approach in HCC, using PSMA-targeted compounds radiolabeled with beta- or alpha-emitting radionuclides, and simultaneously examine the crossfire effect and radiotoxicity of beta emitters in the healthy liver tissue.

A previous systematic review published by Dondi et al. investigated the role of PSMA-targeting radiopharmaceuticals in detecting HCC lesions [56] and did not provide quantitative analyses; furthermore, it included less original articles and some case reports. The present study provided an update of the literature search and added quantitative data through a meta-analysis. Moreover, considering the possible source of biases in including case reports, they were excluded from our research strategy.

In a recent review published by our group [37], we partially covered the same topic of this systematic review and meta-analysis. The current article is a systematic review and meta-analysis, which requires a strict methodology, including appropriate criteria for literature search, quality assessment, quantitative analysis, evaluation of bias. As a result, more articles on PSMA PET in HCC were included, quantitative data through a meta-analysis were provided, a quality assessment of the included articles was performed, and an evaluation of bias was carried out. Furthermore, more data were provided in the results section and in the discussion compared to the previous article. In other words, the previous article was an exploratory review on PSMA-targeted PET imaging which was useful to design a systematic review and meta-analysis on a more specific topic.

With regard to the limitations and biases of this meta-analysis, a restricted number of studies was available. Furthermore, a verification bias cannot be excluded since the authors utilized a composite reference standard in some studies. Finally, a lesion-based meta-analysis was not performed due to missing data. Overall, the authors suggest updating this evidence-based analysis when more original articles in this field of interest are published to accomplish a more accurate estimation of the diagnostic performance of the index test.

Heterogeneity among the included papers (e.g., on account of differences among the selected population, discrepancies in the execution of the index test, study protocol, and quality) may be a source of bias in a meta-analysis [24]. We found a moderate statistical heterogeneity among the included studies, as I^2^ was 56%. A subsequent subgroup analysis omitting the only study that employed PET/MRI as hybrid imaging compound in some of the included patients [33] was performed and the analysis did not show significant statistical heterogeneity demonstrating that the different hybrid imaging method (PET/CT versus PET/MRI) may be a source of heterogeneity.

Based on the current literature data, more prospective multicenter studies with larger sample sizes on the diagnostic accuracy of PSMA-targeted PET imaging in HCC patients are required as well as studies exploring its role in prediction and assessment of treatment response. Moreover, studies evaluating the impact of PSMA-targeted PET imaging on the management of HCC and cost-effectiveness analyses (comparing a diagnostic approach with or without the index test) are needed to assess its role in the management of HCC patients.

## 5. Conclusions

The qualitative and quantitative data provided by this systematic review and meta-analysis highlighted the emerging role of PET/CT or PET/MRI with PSMA-targeting radiopharmaceuticals for the detection of HCC lesions. Nevertheless, more studies are required to corroborate these findings and to better define the role of PET imaging with PSMA-targeting radiopharmaceuticals in this setting (especially when compared to current reference imaging examinations), establishing specific clinical recommendations on this imaging method.

## Figures and Tables

**Table 1:** General study information.

Authors [Ref.]	Year	Country	Study Design/Number of Involved Centers	Funding Sources
Kesler et al. [28]	2018	Israel	Prospective/Monocentric	None declared
Kuyumcu et al. [29]	2019	Turkey	Prospective/Monocentric	None declared
Kunikowska et al. [30]	2020	Poland	Prospective/Monocentric	None declared
Gündoğan et al. [31]	2021	Turkey	Prospective/Monocentric	None declared
Hirmas et al. [32]	2021	Germany	Retrospective/Monocentric	None declared
Thompson et al. [33]	2022	USA	Prospective/Monocentric	None declared

**Table 2:** Patient key characteristics and clinical settings.

Authors [Ref.]	Sample Size (No. Patients)	Mean/Median Age (Years)	Gender (Male %)	Clinical Setting (No. Patients)	Child-Pugh (No. Patients—Score)	PSMA Staining	Comparative Imaging
Kesler et al. [28]	7	Median: 56	71%	staging (6) restaging (1)	3-A 4-B	NVE	[^18^F]FDG PET/CT; ceCT; MRI
Kuyumcu et al. [29]	19	Mean: 57.8	84%	restaging (19)	n.a.	n.a.	[^18^F]FDG PET/CT
Kunikowska et al. [30]	15	Mean 55.6	87%	staging (10) restaging (5)	14-A 1-B	NVE	ceCT; MRI
Gündoğan et al. [31]	14	Mean: 63.8	93%	staging (12) restaging (2)	n.a	n.a.	[^18^F]FDG PET/CT; MRI
Hirmas et al. [32]	40	Median: 65	85%	staging (27) restaging (13)	33-A 7-B	n.a.	ceCT
Thompson et al. [33]	31	Median: 66	74%	staging (31)	n.a	NVE	ceCT; MRI

Legend: CC = cancer cells; ceCT = contrast enhanced computed tomography; [^18^F]FDG PET/CT = fluorine-18 fluorodeoxyglucose positron emission tomography/computed tomography; MRI = magnetic resonance imaging; n.a = not available; NVE: neovascular endothelium; PSMA = prostate-specific membrane antigen.

**Table 3:** Index test key characteristics.

Authors [Ref.]	Tracer	Hybrid Imaging	Tomograph	Administered Activity	Uptake Time (Minutes)	Image Analysis
Kesler et al. [28]	[^68^Ga]Ga-PSMA-11	PET/CT	Discovery 690 (GE ^®^)	148 MBq	n.a	Qualitative and semi- quantitative (SUV_min_, SUV_max_, TBR)
Kuyumcu et al. [29]	[^68^Ga]Ga-PSMA-11	PET/CT	Biograph TruePont (Siemens ^®^)	2.5 MBq/kg ± 10%	60	Qualitative and semi-quantitative (SUV_max_, TBR)
Kunikowska et al. [30]	[^68^Ga]Ga-PSMA-11	PET/CT	Biograph 64 TruePoint (Siemens ^®^)	2 MBq/kg	60	Qualitative and semi-quantitative (SUV_mean_, SUV_max_, TBR)
Gündoğan et al. [31]	[^68^Ga]Ga-PSMA-11	PET/CT	mCT 20 ultra HD LSO (Siemens ^®^)	2-2.5 MBq/kg	60	Qualitative and semi-quantitative (SUV_max_, TBR)
Hirmas et al. [32]	[^68^Ga]Ga-PSMA-11	PET/CT	Biograph 128 mCT; Biograph Vision (Siemens ^®^)	Median: 112.5 MBq	Median: 78	Qualitative and semi-quantitative (SUV_max_)
Thompson et al. [33]	[^68^Ga]Ga-PSMA-11	PET/CT; PET/MRI	Biograph Vision 600 (Siemens ^®^); Signa (GE ^®^)	185 MBq ± 10%	90 ± 15	Qualitative and semi-quantitative (SUV_mean_, SUV_max_, TBR)

Legend: CT = computed tomography; MBq = megaBecquerel; MRI = magnetic resonance imaging; n.a. = not available; PET = positron emission tomography; PSMA = prostate-specific membrane antigen; SUV = standardized uptake value; TBR = tumor-to-background ratio.

**Table 4:** Results of meta-analysis.

Study	Sample Size	Detection Rate (%)	95% CI	Weight (%)	
				Fixed	Random
Kesler et al. [28]	7	85.714	42.128 to 99.639	6.06	10.26
Kuyumku et al. [29]	19	84.211	60.422 to 96.617	15.15	17.05
Kunikowska et al. [30]	15	100.000	78.198 to 100.000	12.12	15.36
Gündoğan et al. [31]	14	92.857	66.132 to 99.819	11.36	14.87
Hirmas et al. [32]	40	90.000	76.336 to 97.207	31.06	22.03
Thompson et al. [33]	31	67.742	48.627 to 83.318	24.24	20.43
Total (fixed effects)	126	85.118	77.881 to 90.712	100	100
Total (random effects)	126	85.942	75.262 to 93.970	100	100

Legend: CI = confidence interval.

## Data Availability

Not applicable.

## Supplementary Materials

The following supporting information can be downloaded at: https://www.mdpi.com/article/10.3390/ph15111368/s1, Table S1: PRISMA checklist. Table S2: list of excluded studies with reasons.
