# Supplementary material for: Can PSMA-Targeting Radiopharmaceuticals Be Useful for Detecting Hepatocellular Carcinoma Using Positron Emission Tomography? An Updated Systematic Review and Meta-Analysis
Source: Pharmaceuticals (Basel). 2022 Nov 8;15(11):1368. doi: 10.3390/ph15111368 (PMC9699564; doi:10.3390/ph15111368)
Supplement: Supplementary file 1 [file pharmaceuticals-15-01368-s001.zip › Supplementary Table S2.pdf]

Excluded studies	Reason of exclusion
Tracht, M.E.; Tallal, L.; Tracht, D.G. Intrinsic Hepatic Control of Psma Albumin Concentration. *Life Sci* **1967**, *6*, 2621–2628, doi:10.1016/0024-3205(67)90112-9.	Original study not in the field of interest
Nandi, D.; Woodward, E.; Ginsburg, D.B.; Monaco, J.J. Intermediates in the Formation of Mouse 20S Proteasomes: Implications for the Assembly of Precursor Beta Subunits. *EMBO J* **1997**, *16*, 5363–5375, doi:10.1093/emboj/16.17.5363.	Original study not in the field of interest
Lamb, H.M.; Faulds, D. Capromab Pendetide. A Review of Its Use as an Imaging Agent in Prostate Cancer. *Drugs Aging* **1998**, *12*, 293–304, doi:10.2165/00002512-199812040-00004.	Review not in the field of interest
Kelly, W.K.; Osman, I.; Reuter, V.E.; Curley, T.; Heston, W.D.; Nanus, D.M.; Scher, H.I. The Development of Biologic End Points in Patients Treated with Differentiation Agents: An Experience of Retinoids in Prostate Cancer. *Clin Cancer Res* **2000**, *6*, 838–846.	Original study not in the field of interest
Sokoloff, R.L.; Norton, K.C.; Gasior, C.L.; Marker, K.M.; Grauer, L.S. A Dual-Monoclonal Sandwich Assay for Prostate-Specific Membrane Antigen: Levels in Tissues, Seminal Fluid and Urine. Prostate **2000**, *43*, 150–157, doi:10.1002/(sici)1097-0045(20000501)43:2<150::aid-pros10>3.0.co;2-b.	Original study not in the field of interest
Chang, S.S.; Reuter, V.E.; Heston, W.D.; Gaudin, P.B. Metastatic Renal Cell Carcinoma Neovasculature Expresses Prostate-Specific Membrane Antigen. *Urology* **2001**, *57*, 801–805, doi:10.1016/s0090-4295(00)01094-3.	Original study not in the field of interest
Chang, S.S.; Reuter, V.E.; Heston, W.D.; Gaudin, P.B. Comparison of Anti-Prostate-Specific Membrane Antigen Antibodies and Other Immunomarkers in Metastatic Prostate Carcinoma. *Urology* **2001**, 57, 1179–1183, doi:10.1016/s0090-4295(01)00983-9.	Original study not in the field of interest
Schmittgen, T.D.; Zakrajsek, B.A.; Hill, R.E.; Liu, Q.; Reeves, J.J.; Axford, P.D.; Singer, M.J.; Reed, M.W. Expression Pattern of Mouse Homolog of Prostate-Specific Membrane Antigen (FOLH1) in the Transgenic Adenocarcinoma of the Mouse Prostate Model. Prostate 2003, 55, 308–316, doi:10.1002/pros.10241.	Original study not in the field of interest
Schmittgen, T.D.; Zakrajsek, B.A.; Hill, R.E.; Liu, Q.; Reeves, J.J.; Axford, P.D.; Singer, M.J.; Reed, M.W. Expression Pattern of Mouse Homolog of Prostate-Specific Membrane Antigen (FOLH1) in the Transgenic Adenocarcinoma of the Mouse Prostate Model. *Prostate* **2003**, *55*, 308–316, doi:10.1002/pros.10241.	Original study not in the field of interest
Chopra, A. 123I-Labeled (S)-2-(3-((S)-1-Carboxy-5-(3-(4-Iodophenyl) Ureido)Pentyl)Ureido)Pentanedoic Acid. In Molecular Imaging and Contrast Agent Database (MICAD); National Center for Biotechnology Information (US): Bethesda (MD), **2004**.	Book chapter not in the field of interest
Leung, K. Quenched Indocyanine Green-Anti-Prostate-Specific Membrane Antigen Antibody J591. In Molecular Imaging and Contrast Agent Database (MICAD); National Center for Biotechnology Information (US): Bethesda (MD), **2004**.	Book chapter not in the field of interest

Leung, K. Cy7-(3S,7S)-26-Amino-5,13,20-Trioxo-4,6,12,21-Tetraazahexacosane-1,3,7,22-Tetracarboxylic Acid. In Molecular Imaging and Contrast Agent Database (MICAD); National Center for Biotechnology Information (US): Bethesda (MD), **2004**.	Book chapter not in the field of interest
Morris, M.J.; Divgi, C.R.; Pandit-Taskar, N.; Batraki, M.; Warren, N.; Nacca, A.; Smith-Jones, P.; Schwartz, L.; Kelly, W.K.; Slovin, S.; et al. Pilot Trial of Unlabeled and Indium-111-Labeled Anti-Prostate-Specific Membrane Antigen Antibody J591 for Castrate Metastatic Prostate Cancer. Clin Cancer Res **2005**, *11*, 7454–7461, doi:10.1158/1078-0432.CCR-05-0826.	Original study not in the field of interest
Kinoshita, Y.; Kuratsukuri, K.; Landas, S.; Imaida, K.; Rovito, P.M.; Wang, C.Y.; Haas, G.P. Expression of Prostate-Specific Membrane Antigen in Normal and Malignant Human Tissues. *World J Surg* **2006**, *30*, 628–636, doi:10.1007/s00268-005-0544-5.	Original study not in the field of interest
Ikegami, S.; Yamakami, K.; Ono, T.; Sato, M.; Suzuki, S.; Yoshimura, I.; Asano, T.; Hayakawa, M.; Tadakuma, T. Targeting Gene Therapy for Prostate Cancer Cells by Liposomes Complexed with Anti-Prostate-Specific Membrane Antigen Monoclonal Antibody. *Hum Gene Ther* **2006**, *17*, 997–1005, doi:10.1089/hum.2006.17.997.	Original study not in the field of interest
Galsky, M.D.; Eisenberger, M.; Moore-Cooper, S.; Kelly, W.K.; Slovin, S.F.; DeLaCruz, A.; Lee, Y.; Webb, I.J.; Scher, H.I. Phase I Trial of the Prostate-Specific Membrane Antigen-Directed Immunoconjugate MLN2704 in Patients with Progressive Metastatic Castration-Resistant Prostate Cancer. J Clin Oncol **2008**, *26*, 2147–2154, doi:10.1200/JCO.2007.15.0532.	Original study not in the field of interest
Haffner, M.C.; Kronberger, I.E.; Ross, J.S.; Sheehan, C.E.; Zitt, M.; Mühlmann, G.; Ofner, D.; Zelger, B.; Ensinger, C.; Yang, X.J.; et al. Prostate-Specific Membrane Antigen Expression in the Neovasculature of Gastric and Colorectal Cancers. *Hum Pathol* **2009**, *40*, 1754–1761, doi:10.1016/j.humpath.2009.06.003.	Original study not in the field of interest
Chopra, A. 123I-Labeled (S)-2-(3-((S)-1-Carboxy-5-(3-(4-Iodophenyl) Ureido)Pentyl)Ureido)Pentanedoic Acid. In Molecular Imaging and Contrast Agent Database (MICAD); National Center for Biotechnology Information (US): Bethesda (MD), **2004**. Updated in **2009**	Book chapter not in the field of interest
Wilbur, D.S.; Chyan, M.-K.; Hamlin, D.K.; Nguyen, H.; Vessella, R.L. Reagents for Astatination of Biomolecules. 5. Evaluation of Hydrazone Linkers in (211)At- and (125)I-Labeled Closo-Decaborate(2-) Conjugates of Fab′ as a Means of Decreasing Kidney Retention. *Bioconjug Chem* **2011**, *22*, 1089–1102, doi:10.1021/bc1005625.	Original study not in the field of interest
Eder, M.; Schäfer, M.; Bauder-Wüst, U.; Hull, W.-E.; Wängler, C.; Mier, W.; Haberkorn, U.; Eisenhut, M. 68Ga-Complex Lipophilicity and the Targeting Property of a Urea-Based PSMA Inhibitor for	Original study not in the field of interest

PET Imaging. *Bioconjug Chem* **2012**, *23*, 688–697, doi:10.1021/bc200279b.	
Hrkach, J.; Von Hoff, D.; Mukkaram Ali, M.; Andrianova, E.; Auer, J.; Campbell, T.; De Witt, D.; Figa, M.; Figueiredo, M.; Horhota, A.; et al. Preclinical Development and Clinical Translation of a PSMA-Targeted Docetaxel Nanoparticle with a Differentiated Pharmacological Profile. *Sci Transl Med* **2012**, *4*, 128ra39, doi:10.1126/scitranslmed.3003651.ù	Original study not in the field of interest
Liu, J.; Kopečková, P.; Pan, H.; Sima, M.; Bühler, P.; Wolf, P.; Elsässer-Beile, U.; Kopeček, J. Prostate-Cancer-Targeted N-(2-Hydroxypropyl)Methacrylamide Copolymer/Docetaxel Conjugates. *Macromol Biosci* **2012**, *12*, 412–422, doi:10.1002/mabi.201100340.	Original study not in the field of interest
Luo, X.-M.; Liu, J.-Y.; Su, M.-Q.; Hao, X.-K. [Transcriptional activities of tumor-specific survivin promoter and PSMA promoter and enhancer in human prostate cancer: evaluation and comparison]. *Zhonghua Nan Ke Xue* **2012,** *18*, 122–125.	Original study not in the field of interest
Chen, Z.; Penet, M.-F.; Nimmagadda, S.; Li, C.; Banerjee, S.R.; Winnard, P.T.; Artemov, D.; Glunde, K.; Pomper, M.G.; Bhujwalla, Z.M. PSMA-Targeted Theranostic Nanoplex for Prostate Cancer Therapy. *ACS Nano* **2012**, *6*, 7752–7762, doi:10.1021/nn301725w.	Original study not in the field of interest
Afshar-Oromieh, A.; Malcher, A.; Eder, M.; Eisenhut, M.; Linhart, H.G.; Hadaschik, B.A.; Holland-Letz, T.; Giesel, F.L.; Kratochwil, C.; Haufe, S.; et al. PET Imaging with a [68Ga]Gallium-Labelled PSMA Ligand for the Diagnosis of Prostate Cancer: Biodistribution in Humans and First Evaluation of Tumour Lesions. *Eur J Nucl Med Mol Imaging* **2013**, *40*, 486–495, doi:10.1007/s00259-012-2298-2.	Original study not in the field of interest
Barrett, J.A.; Coleman, R.E.; Goldsmith, S.J.; Vallabhajosula, S.; Petry, N.A.; Cho, S.; Armor, T.; Stubbs, J.B.; Maresca, K.P.; Stabin, M.G.; et al. First-in-Man Evaluation of 2 High-Affinity PSMA-Avid Small Molecules for Imaging Prostate Cancer. *J Nucl Med* **2013**, *54*, 380–387, doi:10.2967/jnumed.112.111203.	Original study not in the field of interest
Afshar-Oromieh, A.; Haberkorn, U.; Schlemmer, H.P.; Fenchel, M.; Eder, M.; Eisenhut, M.; Hadaschik, B.A.; Kopp-Schneider, A.; Röthke, M. Comparison of PET/CT and PET/MRI Hybrid Systems Using a 68Ga-Labelled PSMA Ligand for the Diagnosis of Recurrent Prostate Cancer: Initial Experience. *Eur J Nucl Med Mol Imaging* **2014**, *41*, 887–897, doi:10.1007/s00259-013-2660-z.	Original study not in the field of interest
El-Zaria, M.E.; Genady, A.R.; Janzen, N.; Petlura, C.I.; Beckford Vera, D.R.; Valliant, J.F. Preparation and Evaluation of Carborane-Derived Inhibitors of Prostate Specific Membrane Antigen (PSMA). Dalton Trans **2014**, *43*, 4950–4961, doi:10.1039/c3dt53189a.	Original study not in the field of interest

Zechmann, C.M.; Afshar-Oromieh, A.; Armor, T.; Stubbs, J.B.; Mier, W.; Hadaschik, B.; Joyal, J.; Kopka, K.; Debus, J.; Babich, J.W.; et al. Radiation Dosimetry and First Therapy Results with a (124)I/ (131)I-Labeled Small Molecule (MIP-1095) Targeting PSMA for Prostate Cancer Therapy. *Eur J Nucl Med Mol Imaging* **2014**, *41*, 1280–1292, doi:10.1007/s00259-014-2713-y.	Original study not in the field of interest
Huang, S.S.; Wang, X.; Zhang, Y.; Doke, A.; DiFilippo, F.P.; Heston, W.D. Improving the Biodistribution of PSMA-Targeting Tracers with a Highly Negatively Charged Linker. *Prostate* **2014**, *74*, 702–713, doi:10.1002/pros.22789.	Original study not in the field of interest
Castanares, M.A.; Mukherjee, A.; Chowdhury, W.H.; Liu, M.; Chen, Y.; Mease, R.C.; Wang, Y.; Rodriguez, R.; Lupold, S.E.; Pomper, M.G. Evaluation of Prostate-Specific Membrane Antigen as an Imaging Reporter. *J Nucl Med* **2014**, *55*, 805–811, doi:10.2967/jnumed.113.134031.	Original study not in the field of interest
Tolmachev, V.; Malmberg, J.; Estrada, S.; Eriksson, O.; Orlova, A. Development of a 124I-Labeled Version of the Anti-PSMA Monoclonal Antibody Capromab for ImmunoPET Staging of Prostate Cancer: Aspects of Labeling Chemistry and Biodistribution. Int J Oncol **2014**, *44*, 1998–2008, doi:10.3892/ijo.2014.2376.	Original study not in the field of interest
Herrmann, K.; Bluemel, C.; Weineisen, M.; Schottelius, M.; Wester, H.-J.; Czernin, J.; Eberlein, U.; Beykan, S.; Lapa, C.; Riedmiller, H.; et al. Biodistribution and Radiation Dosimetry for a Probe Targeting Prostate-Specific Membrane Antigen for Imaging and Therapy. *J Nucl Med* **2015**, *56*, 855–861, doi:10.2967/jnumed.115.156133.	Original study not in the field of interest
Vallabhajosula, S.; Nikolopoulou, A.; Babich, J.W.; Osborne, J.R.; Tagawa, S.T.; Lipai, I.; Solnes, L.; Maresca, K.P.; Armor, T.; Joyal, J.L.; et al. 99mTc-Labeled Small-Molecule Inhibitors of Prostate-Specific Membrane Antigen: Pharmacokinetics and Biodistribution Studies in Healthy Subjects and Patients with Metastatic Prostate Cancer. *J Nucl Med* **2014**, *55*, 1791–1798, doi:10.2967/jnumed.114.140426.	Original study not in the field of interest
Pandit-Taskar, N.; O'Donoghue, J.A.; Divgi, C.R.; Wills, E.A.; Schwartz, L.; Gönen, M.; Smith-Jones, P.; Bander, N.H.; Scher, H.I.; Larson, S.M.; et al. Indium 111-Labeled J591 Anti-PSMA Antibody for Vascular Targeted Imaging in Progressive Solid Tumors. *EJNMMI Res* **2015**, *5*, 28, doi:10.1186/s13550-015-0104-4.	Original study not in the field of interest
Dietlein, M.; Kobe, C.; Kuhnert, G.; Stockter, S.; Fischer, T.; Schomäcker, K.; Schmidt, M.; Dietlein, F.; Zlatopolskiy, B.D.; Krapf, P.; et al. Comparison of [(18)F]DCFPyL and [(68)Ga]Ga-PSMA-HBED-CC for PSMA-PET Imaging in Patients with Relapsed Prostate Cancer. *Mol Imaging Biol* **2015**, *17*, 575–584, doi:10.1007/s11307-015-0866-0.	Original study not in the field of interest

Weineisen, M.; Schottelius, M.; Simecek, J.; Baum, R.P.; Yildiz, A.; Beykan, S.; Kulkarni, H.R.; Lassmann, M.; Klette, I.; Eiber, M.; et al. 68Ga- and 177Lu-Labeled PSMA I&T: Optimization of a PSMA-Targeted Theranostic Concept and First Proof-of-Concept Human Studies. *J Nucl Med* **2015**, *56*, 1169–1176, doi:10.2967/jnumed.115.158550.	Original study not in the field of interest
Ahmadzadehfar, H.; Rahbar, K.; Kürpig, S.; Bögemann, M.; Claesener, M.; Eppard, E.; Gärtner, F.; Rogenhofer, S.; Schäfers, M.; Essler, M. Early Side Effects and First Results of Radioligand Therapy with (177)Lu-DKFZ-617 PSMA of Castrate-Resistant Metastatic Prostate Cancer: A Two-Centre Study. *EJNMMI Res* **2015**, *5*, 114, doi:10.1186/s13550-015-0114-2.	Original study not in the field of interest
Delker, A.; Fendler, W.P.; Kratochwil, C.; Brunegraf, A.; Gosewisch, A.; Gildehaus, F.J.; Tritschler, S.; Stief, C.G.; Kopka, K.; Haberkorn, U.; et al. Dosimetry for (177)Lu-DKFZ-PSMA-617: A New Radiopharmaceutical for the Treatment of Metastatic Prostate Cancer. *Eur J Nucl Med Mol Imaging* **2016**, *43*, 42–51, doi:10.1007/s00259-015-3174-7.	Original study not in the field of interest
Quynh Doan, N.T.; Christensen, S.B. Thapsigargin, Origin, Chemistry, Structure-Activity Relationships and Prodrug Development. *Curr Pharm Des* **2015**, *21*, 5501–5517, doi:10.2174/1381612821666151002112824.	Review not in the field of interest
Fan, W.; Zhang, Z.; Zhu, Z.; Yang, D.; Chen, X.; Wang, J.; Chen, F.; Song, X. Synthesis and Positron Emission Tomography Evaluation of 18F-Glu-Urea-Lys, a Prostate-Specific Membrane Antigen-Based Imaging Agent for Prostate Cancer. *Oncol Lett* **2015,** *10*, 2299–2302, doi:10.3892/ol.2015.3625.	Original study not in the field of interest
Sasikumar, A.; Joy, A.; Nanabala, R.; Pillai, M.R.A.; Thomas, B.; Vikraman, K.R. (68)Ga-PSMA PET/CT Imaging in Primary Hepatocellular Carcinoma. *Eur J Nucl Med Mol Imaging* **2016**, *43*, 795–796, doi:10.1007/s00259-015-3297-x.	Case report in the field of interest
Cleeren, F.; Lecina, J.; Billaud, E.M.F.; Ahamed, M.; Verbruggen, A.; Bormans, G.M. New Chelators for Low Temperature Al(18)F-Labeling of Biomolecules. *Bioconjug Chem* **2016**, *27*, 790–798, doi:10.1021/acs.bioconjchem.6b00012.	Original study not in the field of interest
Rahbar, K.; Schmidt, M.; Heinzel, A.; Eppard, E.; Bode, A.; Yordanova, A.; Claesener, M.; Ahmadzadehfar, H. Response and Tolerability of a Single Dose of 177Lu-PSMA-617 in Patients with Metastatic Castration-Resistant Prostate Cancer: A Multicenter Retrospective Analysis. *J Nucl Med* **2016**, *57*, 1334–1338, doi:10.2967/jnumed.116.173757.	Original study not in the field of interest

Kimura, H.; Sampei, S.; Matsuoka, D.; Harada, N.; Watanabe, H.; Arimitsu, K.; Ono, M.; Saji, H. Development of (99m)Tc-Labeled Asymmetric Urea Derivatives That Target Prostate-Specific Membrane Antigen for Single-Photon Emission Computed Tomography Imaging. *Bioorg Med Chem* **2016**, *24*, 2251–2256, doi:10.1016/j.bmc.2016.03.051.	Original study not in the field of interest
Langsteger, W.; Rezaee, A.; Loidl, W.; Geinitz, H.S.; Fitz, F.; Steinmair, M.; Broinger, G.; Pallwien-Prettner, L.; Beheshti, M.; Imamovic, L.; et al. 32nd International Austrian Winter Symposium : Zell Am See, the Netherlands. 20-23 January 2016. *EJNMMI Res* **2016**, *6*, 32, doi:10.1186/s13550-016-0168-9.	Collection of symposium abstracts
Pfob, C.H.; Ziegler, S.; Graner, F.P.; Köhner, M.; Schachoff, S.; Blechert, B.; Wester, H.-J.; Scheidhauer, K.; Schwaiger, M.; Maurer, T.; et al. Biodistribution and Radiation Dosimetry of (68)Ga-PSMA HBED CC-a PSMA Specific Probe for PET Imaging of Prostate Cancer. *Eur J Nucl Med Mol Imaging* **2016**, *43*, 1962–1970, doi:10.1007/s00259-016-3424-3.	Original study not in the field of interest
Afshar-Oromieh, A.; Hetzheim, H.; Kübler, W.; Kratochwil, C.; Giesel, F.L.; Hope, T.A.; Eder, M.; Eisenhut, M.; Kopka, K.; Haberkorn, U. Radiation Dosimetry of (68)Ga-PSMA-11 (HBED-CC) and Preliminary Evaluation of Optimal Imaging Timing. *Eur J Nucl Med Mol Imaging* **2016**, *43*, 1611–1620, doi:10.1007/s00259-016-3419-0.	Original study not in the field of interest
Chen, Y.; Lisok, A.; Chatterjee, S.; Wharram, B.; Pullambhatla, M.; Wang, Y.; Sgouros, G.; Mease, R.C.; Pomper, M.G. [(18)F]Fluoroethyl Triazole Substituted PSMA Inhibitor Exhibiting Rapid Normal Organ Clearance. *Bioconjug Chem* **2016**, *27*, 1655–1662, doi:10.1021/acs.bioconjchem.6b00195.	Original study not in the field of interest
Boschi, S.; Lee, J.T.; Beykan, S.; Slavik, R.; Wei, L.; Spick, C.; Eberlein, U.; Buck, A.K.; Lodi, F.; Cicoria, G.; et al. Synthesis and Preclinical Evaluation of an Al18F Radiofluorinated GLU-UREA-LYS(AHX)-HBED-CC PSMA Ligand. *Eur J Nucl Med Mol Imaging* **2016**, *43*, 2122–2130, doi:10.1007/s00259-016-3437-y.	Original study not in the field of interest
Demirci, E.; Sahin, O.E.; Ocak, M.; Akovali, B.; Nematyazar, J.; Kabasakal, L. Normal Distribution Pattern and Physiological Variants of 68Ga-PSMA-11 PET/CT Imaging. *Nucl Med Commun* **2016**, *37*, 1169–1179, doi:10.1097/MNM.0000000000000566.	Original study not in the field of interest
Harada, N.; Kimura, H.; Onoe, S.; Watanabe, H.; Matsuoka, D.; Arimitsu, K.; Ono, M.; Saji, H. Synthesis and Biologic Evaluation of Novel 18F-Labeled Probes Targeting Prostate-Specific Membrane Antigen for PET of Prostate Cancer. *J Nucl Med* **2016**, *57*, 1978–1984, doi:10.2967/jnumed.116.175810.	Original study not in the field of interest

Pandit-Taskar, N.; O'Donoghue, J.A.; Ruan, S.; Lyashchenko, S.K.; Carrasquillo, J.A.; Heller, G.; Martinez, D.F.; Cheal, S.M.; Lewis, J.S.; Fleisher, M.; et al. First-in-Human Imaging with 89Zr-Df-IAB2M Anti-PSMA Minibody in Patients with Metastatic Prostate Cancer: Pharmacokinetics, Biodistribution, Dosimetry, and Lesion Uptake. *J Nucl Med* **2016**, *57*, 1858–1864, doi:10.2967/jnumed.116.176206.	Original study not in the field of interest
Yadav, M.P.; Ballal, S.; Tripathi, M.; Damle, N.A.; Sahoo, R.K.; Seth, A.; Bal, C. 177Lu-DKFZ-PSMA-617 Therapy in Metastatic Castration Resistant Prostate Cancer: Safety, Efficacy, and Quality of Life Assessment. *Eur J Nucl Med Mol Imaging* **2017**, *44*, 81–91, doi:10.1007/s00259-016-3481-7.	Original study not in the field of interest
Ferdinandus, J.; Eppard, E.; Gaertner, F.C.; Kürpig, S.; Fimmers, R.; Yordanova, A.; Hauser, S.; Feldmann, G.; Essler, M.; Ahmadzadehfar, H. Predictors of Response to Radioligand Therapy of Metastatic Castrate-Resistant Prostate Cancer with 177Lu-PSMA-617. *J Nucl Med* **2017**, *58*, 312–319, doi:10.2967/jnumed.116.178228.	Original study not in the field of interest
Taneja, S.; Taneja, R.; Kashyap, V.; Jha, A.; Jena, A. 68Ga-PSMA Uptake in Hepatocellular Carcinoma. *Clin Nucl Med* **2017**, *42*, e69–e70, doi:10.1097/RLU.0000000000001355.	Case report in the field of interest
Okamoto, S.; Thieme, A.; Allmann, J.; D'Alessandria, C.; Maurer, T.; Retz, M.; Tauber, R.; Heck, M.M.; Wester, H.-J.; Tamaki, N.; et al. Radiation Dosimetry for 177Lu-PSMA I&T in Metastatic Castration-Resistant Prostate Cancer: Absorbed Dose in Normal Organs and Tumor Lesions. *J Nucl Med* **2017**, *58*, 445–450, doi:10.2967/jnumed.116.178483.	Original study not in the field of interest
Elri, T.; Aras, M.; Salihoglu, Y.S.; Erdemir, R.U.; Cabuk, M. A Potential Pitfall in the Use of 68Ga-PSMA PET/CT: Anthracosis. *Rev Esp Med Nucl Imagen Mol* **2017**, *36*, 65–66, doi:10.1016/j.remn.2016.06.011.	Case report not in the field of interest
Grubmüller, B.; Baum, R.P.; Capasso, E.; Singh, A.; Ahmadi, Y.; Knoll, P.; Floth, A.; Righi, S.; Zandieh, S.; Meleddu, C.; et al. 64Cu-PSMA-617 PET/CT Imaging of Prostate Adenocarcinoma: First In-Human Studies. *Cancer Biother Radiopharm* **2016**, *31*, 277–286, doi:10.1089/cbr.2015.1964.	Original study not in the field of interest
Hermann, R.M.; Djannatian, M.; Czech, N.; Nitsche, M. Prostate-Specific Membrane Antigen PET/CT: False-Positive Results Due to Sarcoidosis? *Case Rep Oncol* **2016**, *9*, 457–463, doi:10.1159/000447688.	Case report not in the field of interest
X, W.; C, S.; C, S.; M, E.; H, A. Successful Treatment of Hepatic Metastases of Hormone Refractory Prostate Cancer Using Radioligand Therapy With 177Lu-PSMA-617. *Clin nucl med* **2016**, *41*, doi:10.1097/RLU.0000000000001358.	Case report in the field of interest

Bhardwaj, H.; Stephens, M.; Bhatt, M.; Thomas, P.A. Prostate-Specific Membrane Antigen PET/CT Findings for Hepatic Hemangioma. *Clin Nucl Med* **2016**, *41*, 968–969, doi:10.1097/RLU.0000000000001384.	Case report not in the field of interest
Jin, W.; Qin, B.; Chen, Z.; Liu, H.; Barve, A.; Cheng, K. Discovery of PSMA-Specific Peptide Ligands for Targeted Drug Delivery. *Int J Pharm* **2016**, *513*, 138–147, doi:10.1016/j.ijpharm.2016.08.048.	Original study not in the field of interest
Yadav, M.P.; Ballal, S.; Tripathi, M.; Damle, N.A.; Sahoo, R.K.; Seth, A.; Bal, C. Post-Therapeutic Dosimetry of 177Lu-DKFZ-PSMA-617 in the Treatment of Patients with Metastatic Castration-Resistant Prostate Cancer. *Nucl Med Commun* **2017**, *38*, 91–98, doi:10.1097/MNM.0000000000000606.	Original study not in the field of interest
Li, X.; Rowe, S.P.; Leal, J.P.; Gorin, M.A.; Allaf, M.E.; Ross, A.E.; Pienta, K.J.; Lodge, M.A.; Pomper, M.G. Semiquantitative Parameters in PSMA-Targeted PET Imaging with 18F-DCFPyL: Variability in Normal-Organ Uptake. *J Nucl Med* **2017**, *58*, 942–946, doi:10.2967/jnumed.116.179739.	Original study not in the field of interest
Davaa, E.; Lee, J.; Jenjob, R.; Yang, S.-G. MT1-MMP Responsive Doxorubicin Conjugated Poly(Lactic-Co-Glycolic Acid)/Poly(Styrene-Alt-Maleic Anhydride) Core/Shell Microparticles for Intrahepatic Arterial Chemotherapy of Hepatic Cancer. *ACS Appl Mater Interfaces* **2017**, *9*, 71–79, doi:10.1021/acsami.6b08994.	Comment not in the field of interest
Stoykow, C.; Huber-Schumacher, S.; Almanasreh, N.; Jilg, C.; Ruf, J. Strong PSMA Radioligand Uptake by Rectal Carcinoma: Who Put the "S" in PSMA? *Clin Nucl Med* **2017**, *42*, 225–226, doi:10.1097/RLU.0000000000001484.	Case report not in the field of interest
Banerjee, S.R.; Foss, C.A.; Horhota, A.; Pullambhatla, M.; McDonnell, K.; Zale, S.; Pomper, M.G. 111In- and IRDye800CW-Labeled PLA-PEG Nanoparticle for Imaging Prostate-Specific Membrane Antigen-Expressing Tissues. *Biomacromolecules* **2017**, *18*, 201–209, doi:10.1021/acs.biomac.6b01485.	Original study not in the field of interest
Bräuer, A.; Rahbar, K.; Konnert, J.; Bögemann, M.; Stegger, L. Diagnostic Value of Additional 68Ga-PSMA-PET before 223Ra-Dichloride Therapy in Patients with Metastatic Prostate Carcinoma. *Nuklearmedizin* **2017**, *56*, 14–22, doi:10.3413/Nukmed-0846-16-09.	Original study not in the field of interest
Scarpa, L.; Buxbaum, S.; Kendler, D.; Fink, K.; Bektic, J.; Gruber, L.; Decristoforo, C.; Uprimny, C.; Lukas, P.; Horninger, W.; et al. The 68Ga/177Lu Theragnostic Concept in PSMA Targeting of Castration-Resistant Prostate Cancer: Correlation of SUVmax Values and Absorbed Dose Estimates. *Eur J Nucl Med Mol Imaging* **2017**, *44*, 788–800, doi:10.1007/s00259-016-3609-9.	Original study not in the field of interest

Usmani, S.; Ahmed, N.; Marafi, F.; Rasheed, R.; Amanguno, H.G.; Al Kandari, F. Molecular Imaging in Neuroendocrine Differentiation of Prostate Cancer: 68Ga-PSMA Versus 68Ga-DOTA NOC PET-CT. *Clin Nucl Med* **2017**, 42, 410–413, doi:10.1097/RLU.0000000000001618.	Case report not in the field of interest
Dureja, S.; Thakral, P.; Pant, V.; Sen, I. Rare Sites of Metastases in Prostate Cancer Detected on Ga-68 PSMA PET/CT Scan-A Case Series. *Indian J Nucl Med* **2017**, *32*, 13–15, doi:10.4103/0972-3919.198450.	Case series not in the field of interest
Pal, S.; George, J.; Singh, A.N.; Mathur, S.; Dash, N.R.; Garg, P.; Sahni, P.; Chattopadhyay, T.K. Posterior Superior Mesenteric Artery (SMA) First Approach vs. Standard Pancreaticoduodenectomy in Patients with Resectable Periampullary Cancers: A Prospective Comparison Focusing on Circumferential Resection Margins. *J Gastrointest Cancer* **2018**, *49*, 252–259, doi:10.1007/s12029-017-9933-x.	Original study not in the field of interest
McCarthy, M.; Langton, T.; Kumar, D.; Campbell, A. Comparison of PSMA-HBED and PSMA-I&T as Diagnostic Agents in Prostate Carcinoma. *Eur J Nucl Med Mol Imaging* **2017**, *44*, 1455–1462, doi:10.1007/s00259-017-3699-z.	Original study not in the field of interest
Kirchner, J.; Schaarschmidt, B.M.; Sawicki, L.M.; Heusch, P.; Hautzel, H.; Ermert, J.; Rabenalt, R.; Antoch, G.; Buchbender, C. Evaluation of Practical Interpretation Hurdles in 68Ga-PSMA PET/CT in 55 Patients: Physiological Tracer Distribution and Incidental Tracer Uptake. *Clin Nucl Med* 2017, 42, e322–e327, doi:10.1097/RLU.0000000000001672.	Original study not in the field of interest
Cui, C.; Hanyu, M.; Hatori, A.; Zhang, Y.; Xie, L.; Ohya, T.; Fukada, M.; Suzuki, H.; Nagatsu, K.; Jiang, C.; et al. Synthesis and Evaluation of [64Cu]PSMA-617 Targeted for Prostate-Specific Membrane Antigen in Prostate Cancer. *Am J Nucl Med Mol Imaging* **2017**, *7*, 40–52.	Original study not in the field of interest
Santos-Cuevas, C.; Davanzo, J.; Ferro-Flores, G.; García-Pérez, F.O.; Ocampo-García, B.; Ignacio-Alvarez, E.; Gómez-Argumosa, E.; Pedraza-López, M. 99mTc-Labeled PSMA Inhibitor: Biokinetics and Radiation Dosimetry in Healthy Subjects and Imaging of Prostate Cancer Tumors in Patients. *Nucl Med Biol* **2017**, *52*, 1–6, doi:10.1016/j.nucmedbio.2017.05.005.	Original study not in the field of interest
Kranzbühler, B.; Tran, S.; Zilli, T.; Burger, I.A. 68Ga-PSMA PET/MR-Positive Peritoneal Metastasis in the Falciform Ligament in Recurrent Prostate Cancer. *Clin Nucl Med* **2017**, *42*, e388–e389, doi:10.1097/RLU.0000000000001703.	Case report not in the field of interest

Bouvet, V.; Wuest, M.; Bailey, J.J.; Bergman, C.; Janzen, N.; Valliant, J.F.; Wuest, F. Targeting Prostate-Specific Membrane Antigen (PSMA) with F-18-Labeled Compounds: The Influence of Prosthetic Groups on Tumor Uptake and Clearance Profile. *Mol Imaging Biol* **2017**, *19*, 923–932, doi:10.1007/s11307-017-1102-x.	Original study not in the field of interest
Baranski, A.-C.; Schäfer, M.; Bauder-Wüst, U.; Wacker, A.; Schmidt, J.; Liolios, C.; Mier, W.; Haberkorn, U.; Eisenhut, M.; Kopka, K.; et al. Improving the Imaging Contrast of 68Ga-PSMA-11 by Targeted Linker Design: Charged Spacer Moieties Enhance the Pharmacokinetic Properties. *Bioconjug Chem* **2017**, 28, 2485–2492, doi:10.1021/acs.bioconjchem.7b00458.	Original study not in the field of interest
Alipour, R.; Gupta, S.; Trethewey, S. 68Ga-PSMA Uptake in Combined Hepatocellular Cholangiocarcinoma With Skeletal Metastases. *Clin Nucl Med* **2017**, *42*, e452–e453, doi:10.1097/RLU.0000000000001771.	Case report not in the field of interest
Sanli, Y.; Kuyumcu, S.; Sanli, O.; Buyukkaya, F.; İribaş, A.; Alcin, G.; Darendeliler, E.; Ozluk, Y.; Yildiz, S.O.; Turkmen, C. Relationships between Serum PSA Levels, Gleason Scores and Results of 68Ga-PSMAPET/CT in Patients with Recurrent Prostate Cancer. *Ann Nucl Med* **2017**, *31*, 709–717, doi:10.1007/s12149-017-1207-y.	Original study not in the field of interest
Gaertner, F.C.; Halabi, K.; Ahmadzadehfar, H.; Kürpig, S.; Eppard, E.; Kotsikopoulos, C.; Liakos, N.; Bundschuh, R.A.; Strunk, H.; Essler, M. Uptake of PSMA-Ligands in Normal Tissues Is Dependent on Tumor Load in Patients with Prostate Cancer. *Oncotarget* **2017**, *8*, 55094–55103, doi:10.18632/oncotarget.19049.	Original study not in the field of interest
Tulsyan, S.; Das, C.J.; Tripathi, M.; Seth, A.; Kumar, R.; Bal, C. Comparison of 68Ga-PSMA PET/CT and Multiparametric MRI for Staging of High-Risk Prostate Cancer68Ga-PSMA PET and MRI in Prostate Cancer. *Nucl Med Commun* **2017**, *38*, 1094–1102, doi:10.1097/MNM.0000000000000749.	Original study not in the field of interest
Eppard, E.; de la Fuente, A.; Benešová, M.; Khawar, A.; Bundschuh, R.A.; Gärtner, F.C.; Kreppel, B.; Kopka, K.; Essler, M.; Rösch, F. Clinical Translation and First In-Human Use of [44Sc]Sc-PSMA-617 for PET Imaging of Metastasized Castrate-Resistant Prostate Cancer. *Theranostics* **2017**, *7*, 4359–4369, doi:10.7150/thno.20586.	Original study not in the field of interest
Sjögreen Gleisner, K.; Spezi, E.; Solny, P.; Gabina, P.M.; Cicone, F.; Stokke, C.; Chiesa, C.; Paphiti, M.; Brans, B.; Sandström, M.; et al. Variations in the Practice of Molecular Radiotherapy and Implementation of Dosimetry: Results from a European Survey. *EJNMMI Phys* **2017**, *4*, 28, doi:10.1186/s40658-017-0193-4.	Survey not in the field of interest
Ahmadzadehfar, H.; Schlolaut, S.; Fimmers, R.; Yordanova, A.; Hirzebruch, S.; Schlenkhoff, C.; Gaertner, F.C.; Awang, Z.H.; Hauser, S.; Essler, M. Predictors of Overall Survival in Metastatic Castration-Resistant Prostate Cancer Patients Receiving [177Lu]Lu-PSMA-617 Radioligand Therapy. *Oncotarget* **2017**, *8*, 103108–103116, doi:10.18632/oncotarget.21600.	Original study not in the field of interest

Kratochwil, C.; Schmidt, K.; Afshar-Oromieh, A.; Bruchertseifer, F.; Rathke, H.; Morgenstern, A.; Haberkorn, U.; Giesel, F.L. Targeted Alpha Therapy of MCRPC: Dosimetry Estimate of 213Bismuth-PSMA-617. *Eur J Nucl Med Mol Imaging* **2018**, *45*, 31–37, doi:10.1007/s00259-017-3817-y.	Original study not in the field of interest
Hofman, M.S.; Eu, P.; Jackson, P.; Hong, E.; Binns, D.; Iravani, A.; Murphy, D.; Mitchell, C.; Siva, S.; Hicks, R.J.; et al. Cold Kit for Prostate-Specific Membrane Antigen (PSMA) PET Imaging: Phase 1 Study of 68Ga-Tris(Hydroxypyridinone)-PSMA PET/CT in Patients with Prostate Cancer. *J Nucl Med* **2018**, *59*, 625–631, doi:10.2967/jnumed.117.199554.	Original study not in the field of interest
Freitag, M.T.; Kesch, C.; Cardinale, J.; Flechsig, P.; Floca, R.; Eiber, M.; Bonekamp, D.; Radtke, J.P.; Kratochwil, C.; Kopka, K.; et al. Simultaneous Whole-Body 18F-PSMA-1007-PET/MRI with Integrated High-Resolution Multiparametric Imaging of the Prostatic Fossa for Comprehensive Oncological Staging of Patients with Prostate Cancer: A Pilot Study. *Eur J Nucl Med Mol Imaging* **2018**, *45*, 340–347, doi:10.1007/s00259-017-3854-6.	Original study not in the field of interest
Laurens, S.T.; Witjes, F.; Janssen, M.; Flucke, U.; Gottardt, M. 68Ga-Prostate-Specific Membrane Antigen Uptake in Gastrointestinal Stromal Tumor. *Clin Nucl Med* **2018**, *43*, 60–61, doi:10.1097/RLU.0000000000001902.	Case report not in the field of interest
Jochumsen, M.R.; Gormsen, L.C.; Nielsen, G.L. 68Ga-PSMA Avid Primary Adenocarcinoma of the Lung With Complementary Low 18F-FDG Uptake. *Clin Nucl Med* **2018**, *43*, 117–119, doi:10.1097/RLU.0000000000001935.	Case report not in the field of interest
Giesel, F.L.; Will, L.; Lawal, I.; Lengana, T.; Kratochwil, C.; Vorster, M.; Neels, O.; Reyneke, F.; Haberkon, U.; Kopka, K.; et al. Intraindividual Comparison of 18F-PSMA-1007 and 18F-DCFPyL PET/CT in the Prospective Evaluation of Patients with Newly Diagnosed Prostate Carcinoma: A Pilot Study. *J Nucl Med* **2018**, *59*, 1076–1080, doi:10.2967/jnumed.117.204669.	Original study not in the field of interest
Derlin, T.; Schmuck, S.; Juhl, C.; Teichert, S.; Zörgiebel, J.; Wester, H.-J.; Schneefeld, S.M.; Walte, A.C.A.; Thackeray, J.T.; Ross, T.L.; et al. Imaging Characteristics and First Experience of [68Ga]THP-PSMA, a Novel Probe for Rapid Kit-Based Ga-68 Labeling and PET Imaging: Comparative Analysis with [68Ga]PSMA I&T. *Mol Imaging Biol* **2018**, *20*, 650–658, doi:10.1007/s11307-018-1160-8.	Original study not in the field of interest
Kwon, Y.-D.; Chung, H.-J.; Lee, S.J.; Lee, S.-H.; Jeong, B.-H.; Kim, H.-K. Synthesis of Novel Multivalent Fluorescent Inhibitors with High Affinity to Prostate Cancer and Their Biological Evaluation. *Bioorg Med Chem Lett* **2018**, *28*, 572–576, doi:10.1016/j.bmcl.2018.01.047.	Original study not in the field of interest

Vadi, S.K.; Kumar, R.; Mittal, B.R.; Parihar, A.S.; Singh, S.K. Unusual Case of Diffuse Penile Metastasis of Prostate Cancer on 68Ga PSMA PET/CT Imaging and 177Lu PSMA Posttherapy Scintigraphy. *Clin Nucl Med* **2018**, *43*, 276–278, doi:10.1097/RLU.0000000000002001.	Case report not in the field of interest
Khawar, A.; Eppard, E.; Sinnes, J.P.; Roesch, F.; Ahmadzadehfar, H.; Kürpig, S.; Meisenheimer, M.; Gaertner, F.C.; Essler, M.; Bundschuh, R.A. [44Sc]Sc-PSMA-617 Biodistribution and Dosimetry in Patients With Metastatic Castration-Resistant Prostate Carcinoma. *Clin Nucl Med* **2018**, *43*, 323–330, doi:10.1097/RLU.0000000000002003.	Original study not in the field of interest
Wondergem, M.; van der Zant, F.M.; Vlottes, P.W.; Knol, R.J.J. Effects of Fasting on 18F-DCFPyL Uptake in Prostate Cancer Lesions and Tissues with Known High Physiologic Uptake. *J Nucl Med* **2018**, *59*, 1081–1084, doi:10.2967/jnumed.117.207316.	Original study not in the field of interest
Rahbar, K.; Afshar-Oromieh, A.; Bögemann, M.; Wagner, S.; Schäfers, M.; Stegger, L.; Weckesser, M. 18F-PSMA-1007 PET/CT at 60 and 120 Minutes in Patients with Prostate Cancer: Biodistribution, Tumour Detection and Activity Kinetics. *Eur J Nucl Med Mol Imaging* **2018**, *45*, 1329–1334, doi:10.1007/s00259-018-3989-0.	Original study not in the field of interest
Arora, S.; Damle, N.A.; Aggarwal, S.; Passah, A.; Behera, A.; Arora, G.; Bal, C.; Tripathi, M. Prostate-Specific Membrane Antigen Expression in Adrenocortical Carcinoma on 68Ga-Prostate-Specific Membrane Antigen PET/CT. *Clin Nucl Med* **2018**, *43*, 449–451, doi:10.1097/RLU.0000000000002064.	Case report not in the field of interest
Passah, A.; Arora, S.; Damle, N.A.; Tripathi, M.; Bal, C.; Subudhi, T.K.; Arora, G. 68Ga-Prostate-Specific Membrane Antigen PET/CT in Triple-Negative Breast Cancer. *Clin Nucl Med* **2018**, *43*, 460–461, doi:10.1097/RLU.0000000000002071.	Case report not in the field of interest
Huang, H.L.; Zhen Loh, T.J.; Hoe Chow, P.K. A Case of Well-Differentiated Hepatocellular Carcinoma Identified on Gallium-68 Prostate-Specific Membrane Antigen Positron Emission Tomography/Computed Tomography. *World J Nucl Med* **2018,** *17*, 102–105, doi:10.4103/wjnm.WJNM_11_17.	Case report in the field of interest
Sevcenco, S.; Klingler, H.C.; Eredics, K.; Friedl, A.; Schneeweiss, J.; Knoll, P.; Kunit, T.; Lusuardi, L.; Mirzaei, S. Application of Cu-64 NODAGA-PSMA PET in Prostate Cancer. *Adv Ther* **2018**, *35*, 779–784, doi:10.1007/s12325-018-0711-3.	Original study not in the field of interest
Dostalova, S.; Polanska, H.; Svobodova, M.; Balvan, J.; Krystofova, O.; Haddad, Y.; Krizkova, S.; Masarik, M.; Eckschlager, T.; Stiborova, M.; et al. Prostate-Specific Membrane Antigen-Targeted Site-Directed Antibody-Conjugated Apoferritin Nanovehicle Favorably Influences In Vivo Side Effects of Doxorubicin. *Sci Rep* **2018**, *8*, 8867, doi:10.1038/s41598-018-26772-z.	Original study not in the field of interest

Prabhu, M.; Damle, N.A.; Gupta, R.; Arora, S.; Arunraj, S.T.; Bal, C. Demonstration of 68Ga-Prostate-Specific Membrane Antigen Uptake in Metastatic Pancreatic Neuroendocrine Tumor. *Indian J Nucl Med* **2018**, *33*, 257–258, doi:10.4103/ijnm.IJNM_6_18.	Case report not in the field of interest
Kesavan, M.; Turner, J.H.; Meyrick, D.; Yeo, S.; Cardaci, G.; Lenzo, N.P. Salvage Radiopeptide Therapy of Advanced Castrate-Resistant Prostate Cancer with Lutetium-177-Labeled Prostate-Specific Membrane Antigen: Efficacy and Safety in Routine Practice. *Cancer Biother Radiopharm* **2018**, *33*, 274–281, doi:10.1089/cbr.2017.2403.	Original study not in the field of interest
Komek, H.; Can, C.; Yilmaz, U.; Altindag, S. Prognostic Value of 68 Ga PSMA I&T PET/CT SUV Parameters on Survival Outcome in Advanced Prostat Cancer. *Ann Nucl Med* **2018**, *32*, 542–552, doi:10.1007/s12149-018-1277-5.	Original study not in the field of interest
Patro, K.C.; Palla, M.; Kashyap, R. Unusual Case of Metastatic Intracranial Hemangiopericytoma and Emphasis on Role of 68Ga-PSMA PET in Imaging. *Clin Nucl Med* **2018**, *43*, e331–e333, doi:10.1097/RLU.0000000000002203.	Case report not in the field of interest
Umbricht, C.A.; Benešová, M.; Hasler, R.; Schibli, R.; van der Meulen, N.P.; Müller, C. Design and Preclinical Evaluation of an Albumin-Binding PSMA Ligand for 64Cu-Based PET Imaging. *Mol Pharm* **2018**, *15*, 5556–5564, doi:10.1021/acs.molpharmaceut.8b00712.	Original study not in the field of interest
Mohammadzadeh, M.; Shirmohammadi, M.; Ghojazadeh, M.; Nikniaz, L.; Raeisi, M.; Aghdas, S.A.M. Dendritic Cells Pulsed with Prostate-Specific Membrane Antigen in Metastatic Castration-Resistant Prostate Cancer Patients: A Systematic Review and Meta-Analysis. *Prostate Int* **2018**, *6*, 119–125, doi:10.1016/j.prnil.2018.04.001.	Review not in the field of interest
Santos-Cuevas, C.; Ferro-Flores, G.; García-Pérez, F.O.; Jiménez-Mancilla, N.; Ramírez-Nava, G.; Ocampo-García, B.; Luna-Gutiérrez, M.; Azorín-Vega, E.; Davanzo, J.; Soldevilla-Gallardo, I. 177Lu-DOTA-HYNIC-Lys(Nal)-Urea-Glu: Biokinetics, Dosimetry, and Evaluation in Patients with Advanced Prostate Cancer. *Contrast Media Mol Imaging* **2018**, 5247153, doi:10.1155/2018/5247153.	Original study not in the field of interest
Hammes, J.; Hohberg, M.; Täger, P.; Wild, M.; Zlatopolskiy, B.; Krapf, P.; Neumaier, B.; Schomäcker, K.; Kobe, C.; Schmidt, M.; et al. Uptake in Non-Affected Bone Tissue Does Not Differ between [18F]-DCFPyL and [68Ga]-HBED-CC PSMA PET/CT. *PLoS One* **2018**, *13*, e0209613, doi:10.1371/journal.pone.0209613.	Original study not in the field of interest
Giesel, F.L.; Kratochwil, C.; Lindner, T.; Marschalek, M.M.; Loktev, A.; Lehnert, W.; Debus, J.; Jäger, D.; Flechsig, P.; Altmann, A.; et al. 68Ga-FAPI PET/CT: Biodistribution and Preliminary Dosimetry Estimate of 2 DOTA-Containing FAP-Targeting Agents in Patients with Various Cancers. *J Nucl Med* **2019**, *60*, 386–392, doi:10.2967/jnumed.118.215913.	Original study not in the field of interest

Hohberg, M.; Kobe, C.; Täger, P.; Hammes, J.; Schmidt, M.; Dietlein, F.; Wild, M.; Heidenreich, A.; Drzezga, A.; Dietlein, M. Combined Early and Late [68Ga]PSMA-HBED-CC PET Scans Improve Lesion Detectability in Biochemical Recurrence of Prostate Cancer with Low PSA Levels. *Mol Imaging Biol* **2019**, *21*, 558–566, doi:10.1007/s11307-018-1263-2.	Original study not in the field of interest
Sathekge, M.; Bruchertseifer, F.; Knoesen, O.; Reyneke, F.; Lawal, I.; Lengana, T.; Davis, C.; Mahapane, J.; Corbett, C.; Vorster, M.; et al. 225Ac-PSMA-617 in Chemotherapy-Naive Patients with Advanced Prostate Cancer: A Pilot Study. *Eur J Nucl Med Mol Imaging* **2019**, *46*, 129–138, doi:10.1007/s00259-018-4167-0.	Original study not in the field of interest
Banerjee, S.R.; Kumar, V.; Lisok, A.; Plyku, D.; Nováková, Z.; Brummet, M.; Wharram, B.; Barinka, C.; Hobbs, R.; Pomper, M.G. Evaluation of 111In-DOTA-5D3, a Surrogate SPECT Imaging Agent for Radioimmunotherapy of Prostate-Specific Membrane Antigen. *J Nucl Med* **2019**, *60*, 400–406, doi:10.2967/jnumed.118.214403.	Original study not in the field of interest
Schottelius, M.; Wurzer, A.; Wissmiller, K.; Beck, R.; Koch, M.; Gorpas, D.; Notni, J.; Buckle, T.; van Oosterom, M.N.; Steiger, K.; et al. Synthesis and Preclinical Characterization of the PSMA-Targeted Hybrid Tracer PSMA-I&F for Nuclear and Fluorescence Imaging of Prostate Cancer. *J Nucl Med* **2019**, *60*, 71–78, doi:10.2967/jnumed.118.212720.	Original study not in the field of interest
Schottelius, M.; Wurzer, A.; Wissmiller, K.; Beck, R.; Koch, M.; Gorpas, D.; Notni, J.; Buckle, T.; van Oosterom, M.N.; Steiger, K.; et al. Synthesis and Preclinical Characterization of the PSMA-Targeted Hybrid Tracer PSMA-I&F for Nuclear and Fluorescence Imaging of Prostate Cancer. *J Nucl Med* **2019**, *60*, 71–78, doi:10.2967/jnumed.118.212720.	Original study not in the field of interest
Joraku, A.; Hatano, K.; Kawai, K.; Kandori, S.; Kojima, T.; Fukumitsu, N.; Isobe, T.; Mori, Y.; Sakata, M.; Hara, T.; et al. Phase I/IIa PET Imaging Study with 89zirconium Labeled Anti-PSMA Minibody for Urological Malignancies. *Ann Nucl Med* **2019**, *33*, 119–127, doi:10.1007/s12149-018-1312-6.	Original study not in the field of interest
Emmett, L.; Crumbaker, M.; Ho, B.; Willowson, K.; Eu, P.; Ratnayake, L.; Epstein, R.; Blanksby, A.; Horvath, L.; Guminski, A.; et al. Results of a Prospective Phase 2 Pilot Trial of 177Lu-PSMA-617 Therapy for Metastatic Castration-Resistant Prostate Cancer Including Imaging Predictors of Treatment Response and Patterns of Progression. *Clin Genitourin Cancer* **2019**, *17*, 15–22, doi:10.1016/j.clgc.2018.09.014.	Original study not in the field of interest
Dowling, M.; Samuelson, J.; Fadl-Alla, B.; Pondenis, H.C.; Byrum, M.; Barger, A.M.; Fan, T.M. Overexpression of Prostate Specific Membrane Antigen by Canine Hemangiosarcoma Cells Provides Opportunity for the Molecular Detection of Disease Burdens within	Original study not in the field of interest

Hemorrhagic Body Cavity Effusions. *PLoS One* **2019**, *14*, e0210297, doi:10.1371/journal.pone.0210297.

Dos Santos, J.C.; Schäfer, M.; Bauder-Wüst, U.; Lehnert, W.; Leotta, K.; Morgenstern, A.; Kopka, K.; Haberkorn, U.; Mier, W.; Kratochwil, C. Development and Dosimetry of 203Pb/212Pb-Labelled PSMA Ligands: Bringing "the Lead" into PSMA-Targeted Alpha Therapy? *Eur J Nucl Med Mol Imaging* **2019**, *46*, 1081–1091, doi:10.1007/s00259-018-4220-z.	Original study not in the field of interest
Perez, P.M.; Flavell, R.R.; Kelley, R.K.; Umetsu, S.; Behr, S.C. Heterogeneous Uptake of 18F-FDG and 68Ga-PSMA-11 in Hepatocellular Carcinoma. *Clin Nucl Med* **2019**, *44*, e133–e135, doi:10.1097/RLU.0000000000002452.	Case report in the field of interest
Jansen, B.H.E.; Kramer, G.M.; Cysouw, M.C.F.; Yaqub, M.M.; de Keizer, B.; Lavalaye, J.; Booij, J.; Vargas, H.A.; Morris, M.J.; Vis, A.N.; et al. Healthy Tissue Uptake of 68Ga-Prostate-Specific Membrane Antigen, 18F-DCFPyL, 18F-Fluoromethylcholine, and 18F-Dihydrotestosterone. *J Nucl Med* **2019**, *60*, 1111–1117, doi:10.2967/jnumed.118.222505.	Original study not in the field of interest
Sandgren, K.; Johansson, L.; Axelsson, J.; Jonsson, J.; Ögren, M.; Ögren, M.; Andersson, M.; Strandberg, S.; Nyholm, T.; Riklund, K.; et al. Radiation Dosimetry of [68Ga]PSMA-11 in Low-Risk Prostate Cancer Patients. *EJNMMI Phys* **2019**, *6*, 2, doi:10.1186/s40658-018-0239-2.	Original study not in the field of interest
Kuo, H.-T.; Lepage, M.L.; Lin, K.-S.; Pan, J.; Zhang, Z.; Liu, Z.; Pryyma, A.; Zhang, C.; Merkens, H.; Roxin, A.; et al. One-Step 18F-Labeling and Preclinical Evaluation of Prostate-Specific Membrane Antigen Trifluoroborate Probes for Cancer Imaging. *J Nucl Med* **2019**, *60*, 1160–1166, doi:10.2967/jnumed.118.216598.	Original study not in the field of interest
Pianou, N.K.; Stavrou, P.Z.; Vlontzou, E.; Rondogianni, P.; Exarhos, D.N.; Datseris, I.E. More Advantages in Detecting Bone and Soft Tissue Metastases from Prostate Cancer Using 18F-PSMA PET/CT. *Hell J Nucl Med* **2019**, *22*, 6–9, doi:10.1967/s002449910952.	Editorial not in the field of interest
Gafita, A.; Bieth, M.; Krönke, M.; Tetteh, G.; Navarro, F.; Wang, H.; Günther, E.; Menze, B.; Weber, W.A.; Eiber, M. QPSMA: Semiautomatic Software for Whole-Body Tumor Burden Assessment in Prostate Cancer Using 68Ga-PSMA11 PET/CT. *J Nucl Med* **2019**, *60*, 1277–1283, doi:10.2967/jnumed.118.224055.	Original study not in the field of interest

Khawar, A.; Eppard, E.; Roesch, F.; Ahmadzadehfar, H.; Kürpig, S.; Meisenheimer, M.; Gaertner, F.C.; Essler, M.; Bundschuh, R.A. Preliminary Results of Biodistribution and Dosimetric Analysis of [68Ga]Ga-DOTAZOL: A New Zoledronate-Based Bisphosphonate for PET/CT Diagnosis of Bone Diseases. *Ann Nucl Med* **2019**, *33*, 404–413, doi:10.1007/s12149-019-01348-7.	Original study not in the field of interest
Marafi, F.; Usmani, S.; Esmail, A. 68Ga-Prostate-Specific Membrane Antigen PET/CT in Cholangiocarcinoma: A Potential Biomarker for Targeted Radioligand Therapy? *Clin Nucl Med* **2019**, *44*, e439–e441, doi:10.1097/RLU.0000000000002563.	Case report not in the field of interest
Piron, S.; De Man, K.; Van Laeken, N.; D'Asseler, Y.; Bacher, K.; Kersemans, K.; Ost, P.; Decaestecker, K.; Deseyne, P.; Fonteyne, V.; et al. Radiation Dosimetry and Biodistribution of 18F-PSMA-11 for PET Imaging of Prostate Cancer. *J Nucl Med* **2019**, *60*, 1736–1742, doi:10.2967/jnumed.118.225250.	Original study not in the field of interest
Hoberück, S.; Wunderlich, G.; Michler, E.; Hölscher, T.; Walther, M.; Seppelt, D.; Platzek, I.; Zöphel, K.; Kotzerke, J. Dual-Time-Point 64 Cu-PSMA-617-PET/CT in Patients Suffering from Prostate Cancer. *J Labelled Comp Radiopharm* **2019**, *62*, 523–532, doi:10.1002/jlcr.3745.	Original study not in the field of interest
Wu, J.; Han, D.; Shi, S.; Zhang, Q.; Zheng, G.; Wei, M.; Han, Y.; Li, G.; Yang, F.; Jiao, D.; et al. A Novel Fully Human Antibody Targeting Extracellular Domain of PSMA Inhibits Tumor Growth in Prostate Cancer. *Mol Cancer Ther* **2019**, *18*, 1289–1301, doi:10.1158/1535-7163.MCT-18-1078.	Original study not in the field of interest
Ferreira, G.; Iravani, A.; Hofman, M.S.; Hicks, R.J. Intra-Individual Comparison of 68Ga-PSMA-11 and 18F-DCFPyL Normal-Organ Biodistribution. *Cancer Imaging* **2019**, *19*, 23, doi:10.1186/s40644-019-0211-y.	Original study not in the field of interest
Sathekge, M.; Bruchertseifer, F.; Vorster, M.; Lawal, I.O.; Knoesen, O.; Mahapane, J.; Davis, C.; Reyneke, F.; Maes, A.; Kratochwil, C.; et al. Predictors of Overall and Disease-Free Survival in Metastatic Castration-Resistant Prostate Cancer Patients Receiving 225Ac-PSMA-617 Radioligand Therapy. *J Nucl Med* **2020**, *61*, 62–69, doi:10.2967/jnumed.119.229229.	Original study not in the field of interest
Sathekge, M.; Bruchertseifer, F.; Vorster, M.; Lawal, I.O.; Knoesen, O.; Mahapane, J.; Davis, C.; Reyneke, F.; Maes, A.; Kratochwil, C.; et al. Predictors of Overall and Disease-Free Survival in Metastatic Castration-Resistant Prostate Cancer Patients Receiving 225Ac-PSMA-617 Radioligand Therapy. *J Nucl Med* **2020**, *61*, 62–69, doi:10.2967/jnumed.119.229229.	Original study not in the field of interest

Jiao, D.; Li, Y.; Yang, F.; Han, D.; Wu, J.; Shi, S.; Tian, F.; Guo, Z.; Xi, W.; Li, G.; et al. Expression of Prostate-Specific Membrane Antigen in Tumor-Associated Vasculature Predicts Poor Prognosis in Hepatocellular Carcinoma. *Clin Transl Gastroenterol* **2019**, *10*, 1–7, doi:10.14309/ctg.0000000000000041.	Original study not in the field of interest
Werner, R.A.; Bundschuh, R.A.; Bundschuh, L.; Lapa, C.; Yin, Y.; Javadi, M.S.; Buck, A.K.; Higuchi, T.; Pienta, K.J.; Pomper, M.G.; et al. Semiquantitative Parameters in PSMA-Targeted PET Imaging with [18F]DCFPyL: Impact of Tumor Burden on Normal Organ Uptake. *Mol Imaging Biol* **2020**, *22*, 190–197, doi:10.1007/s11307-019-01375-w.	Original study not in the field of interest
Werner, R.A.; Bundschuh, R.A.; Bundschuh, L.; Lapa, C.; Yin, Y.; Javadi, M.S.; Buck, A.K.; Higuchi, T.; Pienta, K.J.; Pomper, M.G.; et al. Semiquantitative Parameters in PSMA-Targeted PET Imaging with [18F]DCFPyL: Impact of Tumor Burden on Normal Organ Uptake. *Mol Imaging Biol* **2020**, *22*, 190–197, doi:10.1007/s11307-019-01375-w.	Original study not in the field of interest
Zhang, J.; Kulkarni, H.R.; Singh, A.; Baum, R.P. Complete Regression of Lung Metastases in a Patient With Metastatic Castration-Resistant Prostate Cancer Using 177Lu-PSMA Radioligand Therapy. *Clin Nucl Med* **2020**, *45*, e48–e50, doi:10.1097/RLU.0000000000002655.	Case report not in the field of interest
Damjanovic, J.; Janssen, J.-C.; Prasad, V.; Diederichs, G.; Walter, T.; Brenner, W.; Makowski, M.R. 68Ga-PSMA-PET/CT for the Evaluation of Liver Metastases in Patients with Prostate Cancer. *Cancer Imaging* **2019**, *19*, 37, doi:10.1186/s40644-019-0220-x.	Original study not in the field of interest
Mahalingam, D.; Peguero, J.; Cen, P.; Arora, S.P.; Sarantopoulos, J.; Rowe, J.; Allgood, V.; Tubb, B.; Campos, L. A Phase II, Multicenter, Single-Arm Study of Mipsagargin (G-202) as a Second-Line Therapy Following Sorafenib for Adult Patients with Progressive Advanced Hepatocellular Carcinoma. *Cancers (Basel)* **2019**, *11*, E833, doi:10.3390/cancers11060833.	Original study not in the field of interest
Luo, D.; Wang, X.; Zeng, S.; Ramamurthy, G.; Burda, C.; Basilion, J.P. Targeted Gold Nanocluster-Enhanced Radiotherapy of Prostate Cancer. *Small* **2019**, *15*, e1900968, doi:10.1002/smll.201900968.	Original study not in the field of interest
Morsing, A.; Hildebrandt, M.G.; Vilstrup, M.H.; Wallenius, S.E.; Gerke, O.; Petersen, H.; Johansen, A.; Andersen, T.L.; Høilund-Carlsen, P.F. Hybrid PET/MRI in Major Cancers: A Scoping Review. *Eur J Nucl Med Mol Imaging* **2019**, *46*, 2138–2151, doi:10.1007/s00259-019-04402-8.	Review not in the field of interest
Lindström, E.; Velikyan, I.; Regula, N.; Alhuseinalkhudhur, A.; Sundin, A.; Sörensen, J.; Lubberink, M. Regularized Reconstruction of Digital Time-of-Flight 68Ga-PSMA-11 PET/CT for the Detection of Recurrent Disease in Prostate Cancer Patients. *Theranostics* **2019**, *9*, 3476–3484, doi:10.7150/thno.31970.	Original study not in the field of interest

Tolkach, Y.; Goltz, D.; Kremer, A.; Ahmadzadehfar, H.; Bergheim, D.; Essler, M.; Lam, M.; de Keizer, B.; Fischer, H.-P.; Kristiansen, G. Prostate-Specific Membrane Antigen Expression in Hepatocellular Carcinoma: Potential Use for Prognosis and Diagnostic Imaging. *Oncotarget* **2019**, *10*, 4149–4160, doi:10.18632/oncotarget.27024.	Original study not in the field of interest + Case report in the field of interest
Wang, J.; Zheng, C.; Tan, X.; Zheng, A.; Zeng, Y.; Zhang, Z.; Zhang, X.; Liu, X. Sensitive Fluorometric Determination of Glutathione Using Fluorescent Polymer Dots and the Dopamine-Melanin Nanosystem. Mikrochim Acta 2019, 186, 568, doi:10.1007/s00604-019-3675-3.	Original study not in the field of interest
Paschalis, A.; Sheehan, B.; Riisnaes, R.; Rodrigues, D.N.; Gurel, B.; Bertan, C.; Ferreira, A.; Lambros, M.B.K.; Seed, G.; Yuan, W.; et al. Prostate-Specific Membrane Antigen Heterogeneity and DNA Repair Defects in Prostate Cancer. *Eur Urol* **2019**, *76*, 469–478, doi:10.1016/j.eururo.2019.06.030.	Original study not in the field of interest
Hohberg, M.; Kobe, C.; Krapf, P.; Täger, P.; Hammes, J.; Dietlein, F.; Zlatopolskiy, B.D.; Endepols, H.; Wild, M.; Neubauer, S.; et al. Biodistribution and Radiation Dosimetry of [18F]-JK-PSMA-7 as a Novel Prostate-Specific Membrane Antigen-Specific Ligand for PET/CT Imaging of Prostate Cancer. *EJNMMI Res* **2019**, *9*, 66, doi:10.1186/s13550-019-0540-7.	Original study not in the field of interest
Gallyamov, M.; Meyrick, D.; Barley, J.; Lenzo, N. Renal Outcomes of Radioligand Therapy: Experience of 177lutetium-Prostate-Specific Membrane Antigen Ligand Therapy in Metastatic Castrate-Resistant Prostate Cancer. *Clin Kidney J* **2020**, *13*, 1049–1055, doi:10.1093/ckj/sfz101.	Original study not in the field of interest
Green, M.A.; Hutchins, G.D.; Bahler, C.D.; Tann, M.; Mathias, C.J.; Territo, W.; Sims, J.; Polson, H.; Alexoff, D.; Eckelman, W.C.; et al. [68Ga]Ga-P16-093 as a PSMA-Targeted PET Radiopharmaceutical for Detection of Cancer: Initial Evaluation and Comparison with [68Ga]Ga-PSMA-11 in Prostate Cancer Patients Presenting with Biochemical Recurrence. *Mol Imaging Biol* **2020**, *22*, 752–763, doi:10.1007/s11307-019-01421-7.	Original study not in the field of interest
Hoberück, S.; Michler, E.; Wunderlich, G.; Löck, S.; Hölscher, T.; Froehner, M.; Braune, A.; Ivan, P.; Seppelt, D.; Zöphel, K.; et al. 68Ga-RM2 PET in PSMA- Positive and -Negative Prostate Cancer Patients. *Nuklearmedizin* **2019**, *58*, 352–362, doi:10.1055/a-0990-8898.	Original study not in the field of interest
Kurth, J.; Krause, B.J.; Schwarzenböck, S.M.; Bergner, C.; Hakenberg, O.W.; Heuschkel, M. First-in-Human Dosimetry of Gastrin-Releasing Peptide Receptor Antagonist [177Lu]Lu-RM2: A Radiopharmaceutical for the Treatment of Metastatic Castration-Resistant Prostate Cancer. *Eur J Nucl Med Mol Imaging* **2020**, *47*, 123–135, doi:10.1007/s00259-019-04504-3.	Original study not in the field of interest

Aghdam, R.A.; Amoui, M.; Ghodsirad, M.; Khoshbakht, S.; Mofid, B.; Kaghazchi, F.; Tavakoli, M.; Pirayesh, E.; Ahmadzadehfar, H. Efficacy and Safety of 177Lutetium-Prostate-Specific Membrane Antigen Therapy in Metastatic Castration-Resistant Prostate Cancer Patients: First Experience in West Asia - A Prospective Study. *World J Nucl Med* **2019**, *18*, 258–265, doi:10.4103/wjnm.WJNM_66_18.	Original study not in the field of interest
Van de Wiele, C.; Sathekge, M.; de Spiegeleer, B.; de Jonghe, P.J.; Beels, L.; Maes, A. PSMA-Targeting Positron Emission Agents for Imaging Solid Tumors Other Than Non-Prostate Carcinoma: A Systematic Review. *Int J Mol Sci* **2019**, *20*, E4886, doi:10.3390/ijms20194886.	Review in the field of interest
Suman, S.; Parghane, R.V.; Joshi, A.; Prabhash, K.; Bakshi, G.; Talole, S.; Banerjee, S.; Basu, S. Therapeutic Efficacy, Prognostic Variables and Clinical Outcome of 177Lu-PSMA-617 PRLT in Progressive MCRPC Following Multiple Lines of Treatment: Prognostic Implications of High FDG Uptake on Dual Tracer PET-CT Vis-à-Vis Gleason Score in Such Cohort. *Br J Radiol* **2019**, *92*, 20190380, doi:10.1259/bjr.20190380.	Original study not in the field of interest
Seifert, R.; Kessel, K.; Boegemann, M.; Köhler, M.; Roll, W.; Stegger, L.; Weckesser, M.; Rahbar, K. Additional Local Therapy for Liver Metastases in Patients with Metastatic Castration-Resistant Prostate Cancer Receiving Systemic PSMA-Targeted Therapy. *J Nucl Med* **2020**, *61*, 723–728, doi:10.2967/jnumed.119.233429.	Original study not in the field of interest
Dietlein, F.; Kobe, C.; Hohberg, M.; Zlatopolskiy, B.D.; Krapf, P.; Endepols, H.; Täger, P.; Hammes, J.; Heidenreich, A.; Persigehl, T.; et al. Intraindividual Comparison of 18F-PSMA-1007 with Renally Excreted PSMA Ligands for PSMA PET Imaging in Patients with Relapsed Prostate Cancer. *J Nucl Med* **2020**, *61*, 729–734, doi:10.2967/jnumed.119.234898.	Original study not in the field of interest
Rathke, H.; Holland-Letz, T.; Mier, W.; Flechsig, P.; Mavriopoulou, E.; Röhrich, M.; Kopka, K.; Hohenfellner, M.; Giesel, F.L.; Haberkorn, U.; et al. Response Prediction of 177Lu-PSMA-617 Radioligand Therapy Using Prostate-Specific Antigen, Chromogranin A, and Lactate Dehydrogenase. *J Nucl Med* **2020**, *61*, 689–695, doi:10.2967/jnumed.119.231431.	Original study not in the field of interest
Kunikowska, J.; Kuliński, R.; Muylle, K.; Koziara, H.; Królicki, L. 68Ga-Prostate-Specific Membrane Antigen-11 PET/CT: A New Imaging Option for Recurrent Glioblastoma Multiforme? *Clin Nucl Med* **2020**, *45*, 11–18, doi:10.1097/RLU.0000000000002806.	Original study not in the field of interest
Chen, F.; Ma, K.; Zhang, L.; Madajewski, B.; Turker, M.Z.; Gallazzi, F.; Cruickshank, K.; Zhang, X.; Jenjitranant, P.; Touijer, K.A.; et al. Ultrasmall Renally Clearable Silica Nanoparticles Target Prostate Cancer. *ACS Appl Mater Interfaces* **2019**, *11*, 43879–43887, doi:10.1021/acsami.9b15195.	Original study not in the field of interest

Kumar, R.; Mittal, B.R.; Bhattacharya, A.; Vadi, S.K.; Singh, H.; Bal, A.; Shukla, J.; Singh, H.; Sharma, V.; Sood, A.; et al. Positron Emission Tomography/Computed Tomography Guided Percutaneous Biopsies of Ga-68 Avid Lesions Using an Automated Robotic *Arm. Diagn Interv Imaging* **2020**, *101*, 157–167, doi:10.1016/j.diii.2019.10.006.	Original study not in the field of interest
Maffey-Steffan, J.; Scarpa, L.; Svirydenka, A.; Nilica, B.; Mair, C.; Buxbaum, S.; Bektic, J.; von Guggenberg, E.; Uprimny, C.; Horninger, W.; et al. The 68Ga/177Lu-Theragnostic Concept in PSMA-Targeting of Metastatic Castration-Resistant Prostate Cancer: Impact of Post-Therapeutic Whole-Body Scintigraphy in the Follow-Up. *Eur J Nucl Med Mol Imaging* **2020**, *47*, 695–712, doi:10.1007/s00259-019-04583-2.	Original study not in the field of interest
Gafita, A.; Calais, J.; Franz, C.; Rauscher, I.; Wang, H.; Roberstson, A.; Czernin, J.; Weber, W.A.; Eiber, M. Evaluation of SUV Normalized by Lean Body Mass (SUL) in 68Ga-PSMA11 PET/CT: A Bi-Centric Analysis. *EJNMMI Res* **2019**, *9*, 103, doi:10.1186/s13550-019-0572-z.	Original study not in the field of interest
Hartrampf, P.E.; Seitz, A.K.; Krebs, M.; Buck, A.K.; Lapa, C. False-Negative 18F-PSMA-1007 PET/CT in Metastatic Prostate Cancer Related to High Physiologic Liver Uptake. *Eur J Nucl Med Mol Imaging* **2020**, *47*, 2044–2046, doi:10.1007/s00259-019-04645-5.	Case report not in the field of interest
Rosar, F.; Dewes, S.; Ries, M.; Schaefer, A.; Khreish, F.; Maus, S.; Bohnenberger, H.; Linxweiler, J.; Bartholomä, M.; Ohlmann, C.; et al. New Insights in the Paradigm of Upregulation of Tumoral PSMA Expression by Androgen Receptor Blockade: Enzalutamide Induces PSMA Upregulation in Castration-Resistant Prostate Cancer Even in Patients Having Previously Progressed on Enzalutamide. *Eur J Nucl Med Mol Imaging* **2020**, *47*, 687–694, doi:10.1007/s00259-019-04674-0.	Original study not in the field of interest
Das, J.; Ray, S.; Tapadia, R.; Midha, D.; Mallick, I. Prostate-Specific Membrane Antigen-Expressing Hepatic Lesion: Metastatic or Hepatocellular Carcinoma. *Indian J Nucl Med* **2020**, *35*, 58–60, doi:10.4103/ijnm.IJNM_145_19.	Case report in the field of interest
Qureshi, P.A.A.A.; Asghar, N.; Bashir, H.; Niazi, I.K.; Akhtar, N. The Hot Pleura: Isolated Pleural Metastases From Renal Cell Carcinoma. *Clin Nucl Med* **2020**, *45*, 211–213, doi:10.1097/RLU.0000000000002909.	Case report not in the field of interest
Erhamamci, S.; Aslan, N. Primary Hepatocellular Carcinoma With Intense 68Ga-PSMA Uptake But Slight 18F-FDG Uptake on PET/CT Imaging. *Clin Nucl Med* **2020**, *45*, e176–e177, doi:10.1097/RLU.0000000000002922.	Case report in the field of interest

Yao, X.; Zha, Z.; Ploessl, K.; Choi, S.R.; Zhao, R.; Alexoff, D.; Zhu, L.; Kung, H.F. Synthesis and Evaluation of Novel Radioiodinated PSMA Targeting Ligands for Potential Radiotherapy of Prostate Cancer. *Bioorg Med Chem* **2020**, *28*, 115319, doi:10.1016/j.bmc.2020.115319.	Original study not in the field of interest
Potemkin, R.; Strauch, B.; Kuwert, T.; Prante, O.; Maschauer, S. Development of 18F-Fluoroglycosylated PSMA-Ligands with Improved Renal Clearance Behavior. *Mol Pharm* **2020**, *17*, 933–943, doi:10.1021/acs.molpharmaceut.9b01179.	Original study not in the field of interest
Liu, C.; Liu, T.; Zhang, Z.; Zhang, N.; Du, P.; Yang, Y.; Liu, Y.; Yu, W.; Li, N.; Gorin, M.A.; et al. 68Ga-PSMA PET/CT Combined with PET/Ultrasound-Guided Prostate Biopsy Can Diagnose Clinically Significant Prostate Cancer in Men with Previous Negative Biopsy Results. *J Nucl Med* **2020**, *61*, 1314–1319, doi:10.2967/jnumed.119.235333.	Original study not in the field of interest
Seifert, R.; Kessel, K.; Schlack, K.; Weckesser, M.; Bögemann, M.; Rahbar, K. Radioligand Therapy Using [177Lu]Lu-PSMA-617 in MCRPC: A Pre-VISION Single-Center Analysis. *Eur J Nucl Med Mol Imaging* **2020**, *47*, 2106–2112, doi:10.1007/s00259-020-04703-3.	Original study not in the field of interest
Kurash, M.M.; Gill, R.; Khairulin, M.; Harbosh, H.; Keidar, Z. 68Ga-Labeled PSMA-11 (68Ga-IsoPROtrace-11) Synthesized with Ready to Use Kit: Normal Biodistribution and Uptake Characteristics of Tumour Lesions. *Sci Rep* **2020**, *10*, 3109, doi:10.1038/s41598-020-60099-y.	Original study not in the field of interest
van Boxtel, W.; Lütje, S.; van Engen-van Grunsven, I.C.H.; Verhaegh, G.W.; Schalken, J.A.; Jonker, M.A.; Nagarajah, J.; Gotthardt, M.; van Herpen, C.M.L. 68Ga-PSMA-HBED-CC PET/CT Imaging for Adenoid Cystic Carcinoma and Salivary Duct Carcinoma: A Phase 2 Imaging Study. *Theranostics* **2020**, *10*, 2273–2283, doi:10.7150/thno.38501.	Original study not in the field of interest
Ferdinandus, J.; Violet, J.; Sandhu, S.; Hicks, R.J.; Ravi Kumar, A.S.; Iravani, A.; Kong, G.; Akhurst, T.; Thang, S.P.; Murphy, D.G.; et al. Prognostic Biomarkers in Men with Metastatic Castration-Resistant Prostate Cancer Receiving [177Lu]-PSMA-617. *Eur J Nucl Med Mol Imaging* **2020**, *47*, 2322–2327, doi:10.1007/s00259-020-04723-z.	Original study not in the field of interest
de Vries, L.H.; Lodewijk, L.; Braat, A.J.A.T.; Krijger, G.C.; Valk, G.D.; Lam, M.G.E.H.; Borel Rinkes, I.H.M.; Vriens, M.R.; de Keizer, B. 68Ga-PSMA PET/CT in Radioactive Iodine-Refractory Differentiated Thyroid Cancer and First Treatment Results with 177Lu-PSMA-617. *EJNMMI Res* **2020**, *10*, 18, doi:10.1186/s13550-020-0610-x.	Original study not in the field of interest

Assadi, M.; Rezaei, S.; Jafari, E.; Rekabpour, S.J.; Ravanbod, M.R.; Zohrabi, F.; Amini, A.; Keshmiri, S.; Dadgar, H.; Ahmadzadehfar, H. Potential Application of Lutetium-177-Labeled Prostate-Specific Membrane Antigen-617 Radioligand Therapy for Metastatic Castration-Resistant Prostate Cancer in a Limited Resource Environment: Initial Clinical Experience after 2 Years. *World J Nucl Med* **2020**, *19*, 15–20, doi:10.4103/wjnm.WJNM_20_19.	Original study not in the field of interest
Vats, K.; Agrawal, K.; Sharma, R.; Sarma, H.D.; Satpati, D.; Dash, A. Preparation and Clinical Translation of 99mTc-PSMA-11 for SPECT Imaging of Prostate Cancer. *Medchemcomm* **2019**, *10*, 2111–2117, doi:10.1039/c9md00401g.	Original study not in the field of interest
Stuparu, A.D.; Meyer, C.A.L.; Evans-Axelsson, S.L.; Lückerath, K.; Wei, L.H.; Kim, W.; Poddar, S.; Mona, C.E.; Dahlbom, M.; Girgis, M.D.; et al. Targeted Alpha Therapy in a Systemic Mouse Model of Prostate Cancer - a Feasibility Study. *Theranostics* **2020**, *10*, 2612–2620, doi:10.7150/thno.42228.	Original study not in the field of interest
Tanaka, T.; Yang, M.; Froemming, A.T.; Bryce, A.H.; Inai, R.; Kanazawa, S.; Kawashima, A. Current Imaging Techniques for and Imaging Spectrum of Prostate Cancer Recurrence and Metastasis: A Pictorial Review. *Radiographics* **2020**, *40*, 709–726, doi:10.1148/rg.2020190121.	Review not in the field of interest
Chiu, L.W.; Lawhn-Heath, C.; Behr, S.C.; Juarez, R.; Perez, P.M.; Lobach, I.; Bucknor, M.D.; Hope, T.A.; Flavell, R.R. Factors Predicting Metastatic Disease in 68Ga-PSMA-11 PET-Positive Osseous Lesions in Prostate Cancer. *J Nucl Med* **2020**, *61*, 1779–1785, doi:10.2967/jnumed.119.241174.	Original study not in the field of interest
von Eyben, F.E.; Kulkarni, H.R.; Baum, R.P. Metastatic Extent Predicts Survival as Patients with Metastatic Castration-Resistant Prostate Cancer Are Treated with 177Lu-PSMA Radioligand Therapy. *Theranostics* **2020**, *10*, 4900–4902, doi:10.7150/thno.44568.	Editorial not in the field of interest
Hoberück, S.; Driesnack, S.; Seppelt, D.; Michler, E.; Hölscher, T.; Kotzerke, J. Hepatic Vascular Malformation Mimics PSMA-Positive Prostate Cancer Metastasis. *Clin Nucl Med* **2020**, *45*, e283–e284, doi:10.1097/RLU.0000000000003032.	Case report not in the field of interest
Soyluoglu, S.; Durmus-Altun, G. Animal Models for the Evaluation of Theranostic Radiopharmaceuticals. *Curr Radiopharm* **2021**, *14*, 15–22, doi:10.2174/1874471013666200425223428.	Review not in the field of interest
Zang, J.; Liu, Q.; Sui, H.; Wang, R.; Jacobson, O.; Fan, X.; Zhu, Z.; Chen, X. 177Lu-EB-PSMA Radioligand Therapy with Escalating Doses in Patients with Metastatic Castration-Resistant Prostate Cancer. *J Nucl Med* **2020**, *61*, 1772–1778, doi:10.2967/jnumed.120.242263.	Original study not in the field of interest

Khreish, F.; Kochems, N.; Rosar, F.; Sabet, A.; Ries, M.; Maus, S.; Saar, M.; Bartholomä, M.; Ezziddin, S. Response and Outcome of Liver Metastases in Patients with Metastatic Castration-Resistant Prostate Cancer (MCRPC) Undergoing 177Lu-PSMA-617 Radioligand Therapy. *Eur J Nucl Med Mol Imaging* **2021**, *48*, 103–112, doi:10.1007/s00259-020-04828-5.	Original study not in the field of interest
Kamaldeep, null; Wanage, G.; Sahu, S.K.; Maletha, P.; Adnan, A.; Suman, S.; Basu, S.; Das, T.; Banerjee, S. Examining Absorbed Doses of Indigenously Developed 177Lu-PSMA-617 in Metastatic Castration-Resistant Prostate Cancer Patients at Baseline and During Course of Peptide Receptor Radioligand Therapy. *Cancer Biother Radiopharm* **2021**, *36*, 292–304, doi:10.1089/cbr.2020.3640.	Original study not in the field of interest
Ahmadzadehfar, H.; Rahbar, K.; Baum, R.P.; Seifert, R.; Kessel, K.; Bögemann, M.; Kulkarni, H.R.; Zhang, J.; Gerke, C.; Fimmers, R.; et al. Prior Therapies as Prognostic Factors of Overall Survival in Metastatic Castration-Resistant Prostate Cancer Patients Treated with [177Lu]Lu-PSMA-617. A WARMTH Multicenter Study (the 617 Trial). *Eur J Nucl Med Mol Imaging* **2021**, *48*, 113–122, doi:10.1007/s00259-020-04797-9.	Original study not in the field of interest
Harsini, S.; Saprunoff, H.; Alden, T.; Mohammadi, B.; Wilson, D.; Bénard, F. The Effects of Monosodium Glutamate on PSMA Radiotracer Uptake in Men with Recurrent Prostate Cancer: A Prospective, Randomized, Double-Blind, Placebo-Controlled Intraindividual Imaging Study. *J Nucl Med* **2021**, *62*, 81–87, doi:10.2967/jnumed.120.246983.	Original study not in the field of interest
Trägårdh, E.; Minarik, D.; Brolin, G.; Bitzén, U.; Olsson, B.; Oddstig, J. Optimization of [18F]PSMA-1007 PET-CT Using Regularized Reconstruction in Patients with Prostate Cancer. *EJNMMI Phys* **2020**, *7*, 31, doi:10.1186/s40658-020-00298-8.	Original study not in the field of interest
Lee, I.; Lim, I.; Byun, B.H.; Kim, B.I.; Choi, C.W.; Woo, S.-K.; Lee, K.C.; Kang, J.H.; Kil, H.S.; Park, C.; et al. A Microdose Clinical Trial to Evaluate [18F]Florastamin as a Positron Emission Tomography Imaging Agent in Patients with Prostate Cancer. *Eur J Nucl Med Mol Imaging* **2021**, *48*, 95–102, doi:10.1007/s00259-020-04883-y.	Original study not in the field of interest
Carpanese, D.; Ferro-Flores, G.; Ocampo-Garcia, B.; Santos-Cuevas, C.; Salvarese, N.; Figini, M.; Fracasso, G.; De Nardo, L.; Bolzati, C.; Rosato, A.; et al. Development of 177Lu-ScFvD2B as a Potential Immunotheranostic Agent for Tumors Overexpressing the Prostate Specific Membrane Antigen. *Sci Rep* **2020**, *10*, 9313, doi:10.1038/s41598-020-66285-2.	Original study not in the field of interest
Barna, S.; Haug, A.R.; Hartenbach, M.; Rasul, S.; Grubmüller, B.; Kramer, G.; Blaickner, M. Dose Calculations and Dose-Effect Relationships in 177Lu-PSMA I&T Radionuclide Therapy for Metastatic Castration-Resistant Prostate Cancer. *Clin Nucl Med* **2020**, *45*, 661–667, doi:10.1097/RLU.0000000000003157.	Original study not in the field of interest

Seifert, R.; Seitzer, K.; Herrmann, K.; Kessel, K.; Schäfers, M.; Kleesiek, J.; Weckesser, M.; Boegemann, M.; Rahbar, K. Analysis of PSMA Expression and Outcome in Patients with Advanced Prostate Cancer Receiving 177Lu-PSMA-617 Radioligand Therapy. *Theranostics* **2020**, *10*, 7812–7820, doi:10.7150/thno.47251.	Original study not in the field of interest
Chen, W.; Lee, Z.; Awadallah, A.; Zhou, L.; Xin, W. Peritumoral/Vascular Expression of PSMA as a Diagnostic Marker in Hepatic Lesions. *Diagn Pathol* **2020**, *15*, 92, doi:10.1186/s13000-020-00982-4.	Original study not in the field of interest
Chahinian, R.; El-Amine, A.; Matar, S.; Annan, M.; Shamseddine, A.; Haidar, M. 68Ga-Prostate-Specific Membrane Antigen, A Potential Radiopharmaceutical in PET/CT To Detect Primary Cholangiocarcinoma. *Asia Ocean J Nucl Med Biol* **2020**, *8*, 136–140, doi:10.22038/AOJNMB.2020.46939.1314.	Case report not in the field of interest
Nakano, M.; Kuromatsu, R.; Niizeki, T.; Okamura, S.; Iwamoto, H.; Shimose, S.; Shirono, T.; Noda, Y.; Kamachi, N.; Koga, H.; et al. Primary Treatment with Molecular-Targeted Agents for Hepatocellular Carcinoma: A Propensity Score-Matching Analysis. *Hepatol Commun* **2020**, *4*, 1218–1228, doi:10.1002/hep4.1535.	Original study not in the field of interest
Marinova, M.; Alamdar, R.; Ahmadzadehfar, H.; Essler, M.; Attenberger, U.; Mücke, M.; Conrad, R. Improving Quality of Life in Patients with Metastatic Prostate Cancer Following One Cycle of 177Lu-PSMA-617 Radioligand Therapy: A Pilot Study. *Nuklearmedizin* **2020**, *59*, 409–414, doi:10.1055/a-1234-5891.	Original study not in the field of interest
Trujillo-Benítez, D.; Ferro-Flores, G.; Morales-Avila, E.; Jiménez-Mancilla, N.; Ancira-Cortez, A.; Ocampo-García, B.; Santos-Cuevas, C.; Escudero-Castellanos, A.; Luna-Gutiérrez, M.; Azorín-Vega, E. Synthesis and Biochemical Evaluation of Samarium-153 Oxide Nanoparticles Functionalized with IPSMA-Bombesin Heterodimeric Peptide. *J Biomed Nanotechnol* **2020**, *16*, 689–701, doi:10.1166/jbn.2020.2924.	Original study not in the field of interest
Ancira-Cortez, A.; Ferro-Flores, G.; Jiménez-Mancilla, N.; Morales-Avila, E.; Trujillo-Benítez, D.; Ocampo-García, B.; Santos-Cuevas, C.; Escudero-Castellanos, A.; Luna-Gutiérrez, M. Synthesis, Chemical and Biochemical Characterization of Lu2O3-IPSMA Nanoparticles Activated by Neutron Irradiation. *Mater Sci Eng C Mater Biol Appl* **2020**, *117*, 111335, doi:10.1016/j.msec.2020.111335.	Original study not in the field of interest
Zhao, H.; Li, Y.; Hou, S.; Dai, Y.; Lin, C.; Xu, S. Incidental Detection of Primary Hepatocellular Carcinoma on 18F-Prostate-Specific Membrane Antigen-1007 Positron Emission Tomography/Computed Tomography Imaging in a Patient with Prostate Cancer: A Case Report. *Medicine (Baltimore)* **2020**, *99*, e22486, doi:10.1097/MD.0000000000022486.	Case report in the field of interest

Erhamamcı, S.; Aslan, N. Comparative Findings Between 68Ga-PSMA and 18F-FDG PET/CT for Hepatocellular Carcinoma. *Mol Imaging Radionucl Ther* **2020**, *29*, 135–138, doi:10.4274/mirt.galenos.2020.50455.	Case report in the field of interest
Olde Heuvel, J.; de Wit-van der Veen, B.J.; Donswijk, M.L.; Slump, C.H.; Stokkel, M.P.M. Day-to-Day Variability of [68Ga]Ga-PSMA-11 Accumulation in Primary Prostate Cancer: Effects on Tracer Uptake and Visual Interpretation. *EJNMMI Res* **2020**, *10*, 132, doi:10.1186/s13550-020-00708-z.	Original study not in the field of interest
Bilinski, P.; Webb, M. An Exceptional Response to 177LuPSMA Undermined by Neuroendocrine Transformation. *Urol Case Rep* **2021**, *34*, 101467, doi:10.1016/j.eucr.2020.101467.	Case report not in the field of interest
Marafi, F.; Sasikumar, A.; Aldaas, M.; Esmail, A. 18F-PSMA-1007 PET/CT for Initial Staging of Renal Cell Carcinoma in an End-Stage Renal Disease Patient. *Clin Nucl Med* **2021**, *46*, e65–e67, doi:10.1097/RLU.0000000000003354.	Case report not in the field of interest
Ladrón-de-Guevara, D.; Canelo, A.; Piottante, A.; Regonesi, C. False-Positive 18F-Prostate-Specific Membrane Antigen-1007 PET/CT Caused by Hepatic Multifocal Inflammatory Foci. *Clin Nucl Med* **2021**, *46*, e80–e83, doi:10.1097/RLU.0000000000003425.	Case report not in the field of interest
Banerjee, S.R.; Lisok, A.; Minn, I.; Josefsson, A.; Kumar, V.; Brummet, M.; Boinapally, S.; Brayton, C.; Mease, R.C.; Sgouros, G.; et al. Preclinical Evaluation of 213Bi- and 225Ac-Labeled Low-Molecular-Weight Compounds for Radiopharmaceutical Therapy of Prostate Cancer. *J Nucl Med* **2021**, *62*, 980–988, doi:10.2967/jnumed.120.256388.	Original study not in the field of interest
Piron, S.; Verhoeven, J.; Descamps, B.; Kersemans, K.; De Man, K.; Van Laeken, N.; Pieters, L.; Vral, A.; Vanhove, C.; De Vos, F. Intra-Individual Dynamic Comparison of 18F-PSMA-11 and 68Ga-PSMA-11 in LNCaP Xenograft Bearing Mice. *Sci Rep* **2020**, *10*, 21068, doi:10.1038/s41598-020-78273-7.	Original study not in the field of interest
Wurzer, A.; Parzinger, M.; Konrad, M.; Beck, R.; Günther, T.; Felber, V.; Färber, S.; Di Carlo, D.; Wester, H.-J. Preclinical Comparison of Four [18F, NatGa]RhPSMA-7 Isomers: Influence of the Stereoconfiguration on Pharmacokinetics. *EJNMMI Res* **2020**, *10*, 149, doi:10.1186/s13550-020-00740-z.	Original study not in the field of interest
Feuerecker, B.; Tauber, R.; Knorr, K.; Heck, M.; Beheshti, A.; Seidl, C.; Bruchertseifer, F.; Pickhard, A.; Gafita, A.; Kratochwil, C.; et al. Activity and Adverse Events of Actinium-225-PSMA-617 in Advanced Metastatic Castration-Resistant Prostate Cancer After Failure of Lutetium-177-PSMA. *Eur Urol* **2021**, *79*, 343–350, doi:10.1016/j.eururo.2020.11.013.	Original study not in the field of interest
Calabrò, D.; Argalia, G.; Ambrosini, V. Role of PET/CT and Therapy Management of Pancreatic Neuroendocrine Tumors. *Diagnostics (Basel)* **2020**, *10*, E1059, doi:10.3390/diagnostics10121059.	Review not in the field of interest

Sakthivel, P.; Kumar, A.; Arunraj, S.T.; Thakur, K.; Jaiswal, A.S.; Singh, C.A.; Kumar, R. 68Ga-Prostate-Specific Membrane Antigen PET/CT in Sinonasal Glomangiopericytoma-Exploring Theranostic Avenues! *Clin Nucl Med* **2021**, *46*, 340–341, doi:10.1097/RLU.0000000000003467.	Case report not in the field of interest
Rahbar, K.; Afshar-Oromieh, A.; Seifert, R.; Wagner, S.; Schäfers, M.; Bögemann, M.; Weckesser, M. Do Fasting or High Caloric Drinks Affect the Physiological Uptake of Fluorine-18 Prostate-Specific Membrane Antigen-1007 in Liver and Bowel? *World J Nucl Med* **2020**, *19*, 220–223, doi:10.4103/wjnm.WJNM_6_19.	Original study not in the field of interest
Gupta, M.; Karthikeyan, G.; Choudhury, P.S.; Sharma, A.; Singh, A.; Rawal, S. Is 177Lu-PSMA an Effective Treatment Modality for MCRPC Patients with Bone and Visceral Metastasis? *Hell J Nucl Med* **2020**, *23*, 312–320, doi:10.1967/s002449912219.	Original study not in the field of interest
Parihar, A.S.; Chandekar, K.R.; Singh, H.; Sood, A.; Mittal, B.R. Orbital and Brain Metastases on 68Ga-PSMA PET/CT in a Patient with Prostate Carcinoma Refractory to 177Lu-PSMA and 225Ac-PSMA Therapy. *Asia Ocean J Nucl Med Biol* **2021**, *9*, 67–70, doi:10.22038/AOJNMB.2020.50820.1347.	Case report not in the field of interest
Maliha, P.G.; Singerman, J.; Abikhzer, G.; Probst, S. Physiologic Prostate-Specific Membrane Antigen-Targeted 18F-DCFPyL Uptake in the Epididymis Head Newly Appreciated on Digital PET/CT. *Nucl Med Commun* **2021**, *42*, 490–494, doi:10.1097/MNM.0000000000001363.	Original study not in the field of interest
Lee, C.-H.; Lim, I.; Woo, S.-K.; Kim, K.I.; Lee, K.C.; Song, K.; Choi, C.W.; Lim, S.M. The Feasibility of 64Cu-PSMA I&T PET for Prostate Cancer. *Cancer Biother Radiopharm* **2022**, *37*, 417–423, doi:10.1089/cbr.2020.4189.	Original study not in the field of interest
Vlachostergios, P.J.; Niaz, M.J.; Skafida, M.; Mosallaie, S.A.; Thomas, C.; Christos, P.J.; Osborne, J.R.; Molina, A.M.; Nanus, D.M.; Bander, N.H.; et al. Imaging Expression of Prostate-Specific Membrane Antigen and Response to PSMA-Targeted β-Emitting Radionuclide Therapies in Metastatic Castration-Resistant Prostate Cancer. *Prostate* **2021**, *81*, 279–285, doi:10.1002/pros.24104.	Original study not in the field of interest
Chen, L.-X.; Zou, S.-J.; Li, D.; Zhou, J.-Y.; Cheng, Z.-T.; Zhao, J.; Zhu, Y.-L.; Kuang, D.; Zhu, X.-H. Prostate-Specific Membrane Antigen Expression in Hepatocellular Carcinoma, Cholangiocarcinoma, and Liver Cirrhosis. *World J Gastroenterol* **2020**, *26*, 7664–7678, doi:10.3748/wjg.v26.i48.7664.	Original study not in the field of interest
Usmani, S.; Rasheed, R.; Al Kandari, F.; Ahmed, N. Occult Bone Metastases From Hepatocellular Carcinoma Detected on 68Ga-PMSA PET/CT. *Clin Nucl Med* **2021**, *46*, 661–663, doi:10.1097/RLU.0000000000003515.	Case report in the field of interest

Yang, F.-J.; Ai, S.-Y.; Wu, R.; Lv, Y.; Xie, H.-F.; Dong, Y.; Meng, Q.-L.; Wang, F. Impact of Total Variation Regularized Expectation Maximization Reconstruction on the Image Quality of 68Ga-PSMA PET: A Phantom and Patient Study. *Br J Radiol* **2021**, *94*, 20201356, doi:10.1259/bjr.20201356.	Original study not in the field of interest
Hofman, M.S.; Emmett, L.; Sandhu, S.; Iravani, A.; Joshua, A.M.; Goh, J.C.; Pattison, D.A.; Tan, T.H.; Kirkwood, I.D.; Ng, S.; et al. [177Lu]Lu-PSMA-617 versus Cabazitaxel in Patients with Metastatic Castration-Resistant Prostate Cancer (TheraP): A Randomised, Open-Label, Phase 2 Trial. *Lancet* **2021**, *397*, 797–804, doi:10.1016/S0140-6736(21)00237-3.	Original study not in the field of interest
Tuncel, M.; Telli, T.; Tuncalı, M.Ç.; Karabulut, E. Predictive Factors of Tumor Sink Effect: Insights from 177Lu-Prostate-Specific Membrane Antigen Therapy. *Ann Nucl Med* **2021**, *35*, 529–539, doi:10.1007/s12149-021-01593-9.	Original study not in the field of interest
Zhao, J.; Xue, Q.; Chen, X.; You, Z.; Wang, Z.; Yuan, J.; Liu, H.; Hu, L. Evaluation of SUVlean Consistency in FDG and PSMA PET/MR with Dixon-, James-, and Janma-Based Lean Body Mass Correction. *EJNMMI Phys* **2021**, *8*, 17, doi:10.1186/s40658-021-00363-w.	Original study not in the field of interest
Weber, M.; Jentzen, W.; Hofferber, R.; Herrmann, K.; Fendler, W.P.; Conti, M.; Wetter, A.; Kersting, D.; Rischpler, C.; Fragoso Costa, P. Evaluation of [68Ga]Ga-PSMA PET/CT Images Acquired with a Reduced Scan Time Duration in Prostate Cancer Patients Using the Digital Biograph Vision. *EJNMMI Res* **2021**, *11*, 21, doi:10.1186/s13550-021-00765-y.	Original study not in the field of interest
Vlachostergios, P.J.; Niaz, M.J.; Sun, M.; Mosallaie, S.A.; Thomas, C.; Christos, P.J.; Osborne, J.R.; Molina, A.M.; Nanus, D.M.; Bander, N.H.; et al. Prostate-Specific Membrane Antigen Uptake and Survival in Metastatic Castration-Resistant Prostate Cancer. *Front Oncol* **2021**, *11*, 630589, doi:10.3389/fonc.2021.630589.	Original study not in the field of interest
Meyrick, D.; Gallyamov, M.; Sabarimurugan, S.; Falzone, N.; Lenzo, N. Real-World Data Analysis of Efficacy and Survival After Lutetium-177 Labelled PSMA Ligand Therapy in Metastatic Castration-Resistant Prostate Cancer. *Target Oncol* **2021**, *16*, 369–380, doi:10.1007/s11523-021-00801-w.	Original study not in the field of interest
Uprimny, C.; Bayerschmidt, S.; Kroiss, A.S.; Fritz, J.; Nilica, B.; Svirydenka, H.; Decristoforo, C.; von Guggenberg, E.; Horninger, W.; Virgolini, I.J. Early Injection of Furosemide Increases Detection Rate of Local Recurrence in Prostate Cancer Patients with Biochemical Recurrence Referred for 68Ga-PSMA-11 PET/CT. *J Nucl Med* **2021**, *62*, 1550–1557, doi:10.2967/jnumed.120.261866.	Original study not in the field of interest
Eder, A.-C.; Schäfer, M.; Schmidt, J.; Bauder-Wüst, U.; Roscher, M.; Leotta, K.; Haberkorn, U.; Kopka, K.; Eder, M. Rational Linker Design to Accelerate Excretion and Reduce Background Uptake of Peptidomimetic PSMA-Targeting Hybrid Molecules. *J Nucl Med* **2021**, *62*, 1461–1467, doi:10.2967/jnumed.120.248443.	Original study not in the field of interest

Zhang, X.; Wu, Y.; Zeng, Q.; Xie, T.; Yao, S.; Zhang, J.; Cui, M. Synthesis, Preclinical Evaluation, and First-in-Human PET Study of Quinoline-Containing PSMA Tracers with Decreased Renal Excretion. *J Med Chem* **2021**, *64*, 4179–4195, doi:10.1021/acs.jmedchem.1c00117.	Original study not in the field of interest
Kesavan, M.; Meyrick, D.; Gallyamov, M.; Turner, J.H.; Yeo, S.; Cardaci, G.; Lenzo, N.P. Efficacy and Haematologic Toxicity of Palliative Radioligand Therapy of Metastatic Castrate-Resistant Prostate Cancer with Lutetium-177-Labeled Prostate-Specific Membrane Antigen in Heavily Pre-Treated Patients. *Diagnostics (Basel)* **2021**, *11*, 515, doi:10.3390/diagnostics11030515.	Original study not in the field of interest
Veerasuri, S.; Redman, S.; Graham, R.; Meehan, C.; Little, D. Non-Prostate Uptake on 18F-PSMA-1007 PET/CT: A Case of Myeloma. *BJR Case Rep* **2021**, *7*, 20200102, doi:10.1259/bjrcr.20200102.	Case report not in the field of interest
Rosar, F.; Schön, N.; Bohnenberger, H.; Bartholomä, M.; Stemler, T.; Maus, S.; Khreish, F.; Ezziddin, S.; Schaefer-Schuler, A. Comparison of Different Methods for Post-Therapeutic Dosimetry in [177Lu]Lu-PSMA-617 Radioligand Therapy. *EJNMMI Phys* **2021**, *8*, 40, doi:10.1186/s40658-021-00385-4.	Original study not in the field of interest
Canseco-Hernández, O.; Ferro-Flores, G.; Jimenez-Mancilla, N.; Aranda-Lara, L.; Ocampo-Garcia, B.; Trujillo-Benitez, D.; Ancira-Cortés, A.; Morales-Avila, E.; Santos-Cuevas, C. Preparation and Dosimetry Assessment of $166Dy_2O_3/166Ho_2O_3$-IPSMA Nanoparticles for Targeted Hepatocarcinoma Radiotherapy. *J Nanosci Nanotechnol* **2021**, *21*, 5449–5458, doi:10.1166/jnn.2021.19455.	Original study not in the field of interest
Treiber, H.; König, A.; Neesse, A.; Richter, A.; Sahlmann, C.O.; Strauss, A. Liver Enzyme Elevation After 177Lu-PSMA Radioligand Therapy for Metastasized Castration-Resistant Prostate Cancer. *J Nucl Med* **2021**, *62*, 1016–1019, doi:10.2967/jnumed.120.258533.	Case report not in the field of interest
Ahmadzadehfar, H.; Matern, R.; Baum, R.P.; Seifert, R.; Kessel, K.; Bögemann, M.; Kratochwil, C.; Rathke, H.; Ilhan, H.; Svirydenka, H.; et al. The Impact of the Extent of the Bone Involvement on Overall Survival and Toxicity in MCRPC Patients Receiving [177Lu]Lu-PSMA-617: A WARMTH Multicentre Study. *Eur J Nucl Med Mol Imaging* **2021**, *48*, 4067–4076, doi:10.1007/s00259-021-05383-3.	Original study not in the field of interest
Muzaffar, S.; Ahmed, N.; Rahman, U.; Al Kandari, F.; Usmani, S. 68Ga-Prostate-Specific Membrane Antigen Uptake as a Surrogate Biomarker of Neovascularity in Hepatocellular Carcinoma. *Indian J Nucl Med* **2021**, *36*, 90–91, doi:10.4103/ijnm.IJNM_38_20.	Case report in the field of interest
Gafita, A.; Wang, H.; Robertson, A.; Armstrong, W.R.; Zaum, R.; Weber, M.; Yagubbayli, F.; Kratochwil, C.; Grogan, T.R.; Nguyen, K.; et al. Tumor Sink Effect in 68Ga-PSMA-11 PET: Myth or Reality? *J Nucl Med* **2022**, *63*, 226–232, doi:10.2967/jnumed.121.261906.	Original study not in the field of interest

Lankoff, A.; Czerwińska, M.; Walczak, R.; Karczmarczyk, U.; Tomczyk, K.; Brzóska, K.; Fracasso, G.; Garnuszek, P.; Mikołajczak, R.; Kruszewski, M. Design and Evaluation of 223Ra-Labeled and Anti-PSMA Targeted NaA Nanozeolites for Prostate Cancer Therapy-Part II. Toxicity, Pharmacokinetics and Biodistribution. *Int J Mol Sci* **2021**, *22*, 5702, doi:10.3390/ijms22115702.	Original study not in the field of interest
Weitzer, F.; Nazerani-Hooshmand, T.; Aigner, R.M.; Pernthaler, B. Different Appearances of 3 Malignancies in 68Ga-PSMA-11 Versus 18F-FDG PET/CT. *Clin Nucl Med* **2021**, *46*, e358–e359, doi:10.1097/RLU.0000000000003538.	Case report in the field of interest
Mittlmeier, L.M.; Brendel, M.; Beyer, L.; Albert, N.L.; Todica, A.; Zacherl, M.J.; Wenter, V.; Herlemann, A.; Kretschmer, A.; Ledderose, S.T.; et al. Feasibility of Different Tumor Delineation Approaches for 18F-PSMA-1007 PET/CT Imaging in Prostate Cancer Patients. *Front Oncol* **2021**, *11*, 663631, doi:10.3389/fonc.2021.663631.	Original study not in the field of interest
Uijen, M.J.M.; Derks, Y.H.W.; Merkx, R.I.J.; Schilham, M.G.M.; Roosen, J.; Privé, B.M.; van Lith, S. a. M.; van Herpen, C.M.L.; Gotthardt, M.; Heskamp, S.; et al. PSMA Radioligand Therapy for Solid Tumors Other than Prostate Cancer: Background, Opportunities, Challenges, and First Clinical Reports. *Eur J Nucl Med Mol Imaging* **2021**, *48*, 4350–4368, doi:10.1007/s00259-021-05433-w.	Review in the field of interest
Liu, T.; Liu, C.; Zhang, Z.; Zhang, N.; Guo, X.; Xia, L.; Jiang, J.; Xie, Q.; Yan, K.; Rowe, S.P.; et al. 64Cu-PSMA-BCH: A New Radiotracer for Delayed PET Imaging of Prostate Cancer. *Eur J Nucl Med Mol Imaging* **2021**, *48*, 4508–4516, doi:10.1007/s00259-021-05426-9.	Original study not in the field of interest
Lopez-Bujanda, Z.A.; Obradovic, A.; Nirschl, T.R.; Crowley, L.; Macedo, R.; Papachristodoulou, A.; O'Donnell, T.; Laserson, U.; Zarif, J.C.; Reshef, R.; et al. TGM4: An Immunogenic Prostate-Restricted Antigen. *J Immunother Cancer* **2021**, *9*, e001649, doi:10.1136/jitc-2020-001649.	Original study not in the field of interest
Peters, S.M.B.; Privé, B.M.; de Bakker, M.; de Lange, F.; Jentzen, W.; Eek, A.; Muselaers, C.H.J.; Mehra, N.; Witjes, J.A.; Gotthardt, M.; et al. Intra-Therapeutic Dosimetry of [177Lu]Lu-PSMA-617 in Low-Volume Hormone-Sensitive Metastatic Prostate Cancer Patients and Correlation with Treatment Outcome. *Eur J Nucl Med Mol Imaging* **2022**, *49*, 460–469, doi:10.1007/s00259-021-05471-4.	Original study not in the field of interest
Kalshetty, A.; Menon, B.; Rakshit, S.; Bhattacharjee, A.; Basu, S. Correlation of Lesional Uptake Parameters and Ratios with MiPSMA Score and Estimating Normal Physiologic Concentration: An Exploratory Analysis in Metastatic Castration-Resistant Prostatic Carcinoma Patients with 68Ga-PSMA-11 PET/CT. *J Nucl Med Technol* **2021**, *49*, 235–240, doi:10.2967/jnmt.120.261289.	Original study not in the field of interest

Oflas, M.; Ozluk, Y.; Sanli, O.; Ozkan, Z.G.; Kuyumcu, S. 68Ga-PSMA Uptake Patterns of Clear Cell Renal Carcinoma Across Different Histopathological Subtypes. *Clin Nucl Med* **2022**, *47*, e45–e46, doi:10.1097/RLU.0000000000003814.	Case series not in the field of interest
Chatachot, K.; Shiratori, S.; Chaiwatanarat, T.; Khamwan, K. Patient Dosimetry of 177Lu-PSMA I&T in Metastatic Prostate Cancer Treatment: The Experience in Thailand. *Ann Nucl Med* **2021**, *35*, 1193–1202, doi:10.1007/s12149-021-01659-8.	Original study not in the field of interest
Bilgic, S.; Sayman, H.B.; Sager, M.S.; Sonmezoglu, K. A Case of Hepatic Focal Nodular Hyperplasia Mimicking Hepatocellular Carcinoma Identified on Gallium-68-Prostate-Specific Membrane Antigen Positron Emission Tomography/Computed Tomography. *World J Nucl Med* **2021**, *20*, 192–194, doi:10.4103/wjnm.WJNM_108_20.	Case report in the field of interest
Schierz, J.-H.; Sarikaya, I.; Albatineh, A.N.; Sarikaya, A. Assessing the Correlation Between 68Ga-PSMA-11 Renal PET Parameters and Renal Function Tests. *J Nucl Med Technol* **2022**, *50*, 43–48, doi:10.2967/jnmt.121.262462.	Original study not in the field of interest
de Souza, S.P.M.; Tobar, N.; Frasson, F.; Perini, E.A.; de Souza, C.A.; Delamain, M.T.; Ramos, C.D. Head-to-Head Comparison between 68Ga-PSMA and 18F-FDG-PET/CT in Lymphomas: A Preliminary Analysis. *Nucl Med Commun* **2021**, *42*, 1355–1360, doi:10.1097/MNM.0000000000001465.	Original study not in the field of interest
Sergieva, S.; Mangaldgiev, R.; Dimcheva, M.; Nedev, K.; Zahariev, Z.; Robev, B. SPECT-CT Imaging with [99mTc]PSMA-T4 in Patients with Recurrent Prostate Cancer. *Nucl Med Rev Cent East Eur* **2021**, *24*, 70–81, doi:10.5603/NMR.2021.0018.	Original study not in the field of interest
Pattison, D.A.; Debowski, M.; Gulhane, B.; Arnfield, E.G.; Pelecanos, A.M.; Garcia, P.L.; Latter, M.J.; Lin, C.Y.; Roberts, M.J.; Ramsay, S.C.; et al. Prospective Intra-Individual Blinded Comparison of [18F]PSMA-1007 and [68 Ga]Ga-PSMA-11 PET/CT Imaging in Patients with Confirmed Prostate Cancer. *Eur J Nucl Med Mol Imaging* **2022**, *49*, 763–776, doi:10.1007/s00259-021-05520-y.	Original study not in the field of interest
Khreish, F.; Ribbat, K.; Bartholomä, M.; Maus, S.; Stemler, T.; Hierlmeier, I.; Linxweiler, J.; Schreckenberger, M.; Ezziddin, S.; Rosar, F. Value of Combined PET Imaging with [18F]FDG and [68Ga]Ga-PSMA-11 in MCRPC Patients with Worsening Disease during [177Lu]Lu-PSMA-617 RLT. *Cancers (Basel)* **2021**, *13*, 4134, doi:10.3390/cancers13164134.	Original study not in the field of interest
Khreish, F.; Wiessner, M.; Rosar, F.; Ghazal, Z.; Sabet, A.; Maus, S.; Linxweiler, J.; Bartholomä, M.; Ezziddin, S. Response Assessment and Prediction of Progression-Free Survival by 68Ga-PSMA-11 PET/CT Based on Tumor-to-Liver Ratio (TLR) in Patients with MCRPC Undergoing 177Lu-PSMA-617 Radioligand Therapy. *Biomolecules* **2021**, *11*, 1099, doi:10.3390/biom11081099.	Original study not in the field of interest

Johnsson, K.; Brynolfsson, J.; Sahlstedt, H.; Nickols, N.G.; Rettig, M.; Probst, S.; Morris, M.J.; Bjartell, A.; Eiber, M.; Anand, A. Analytical Performance of APROMISE: Automated Anatomic Contextualization, Detection, and Quantification of [18F]DCFPyL (PSMA) Imaging for Standardized Reporting. *Eur J Nucl Med Mol Imaging* **2022**, *49*, 1041–1051, doi:10.1007/s00259-021-05497-8.	Original study not in the field of interest
Farolfi, A.; Telo, S.; Castellucci, P.; Morais de Campos, A.L.; Rosado-de-Castro, P.H.; Altino de Almeida, S.; Artigas, C.; Scarlattei, M.; Leal, A.; Deandreis, D.; et al. Lung Uptake Detected by 68Ga-PSMA-11 PET/CT in Prostate Cancer Patients with SARS-CoV-2: A Case Series. *Am J Nucl Med Mol Imaging* **2021**, *11*, 300–306.	Case series not in the field of interest
Feuerecker, B.; Chantadisai, M.; Allmann, A.; Tauber, R.; Allmann, J.; Steinhelfer, L.; Rauscher, I.; Wurzer, A.; Wester, H.-J.; Weber, W.A.; et al. Pretherapeutic Comparative Dosimetry of 177Lu-RhPSMA-7.3 and 177Lu-PSMA I&T in Patients with Metastatic Castration-Resistant Prostate Cancer. *J Nucl Med* **2022**, *63*, 833–839, doi:10.2967/jnumed.121.262671.	Original study not in the field of interest
Wang, G.; Hong, H.; Zang, J.; Liu, Q.; Jiang, Y.; Fan, X.; Zhu, Z.; Zhu, L.; Kung, H.F. Head-to-Head Comparison of [68 Ga]Ga-P16-093 and [68 Ga]Ga-PSMA-617 in Dynamic PET/CT Evaluation of the Same Group of Recurrent Prostate Cancer Patients. *Eur J Nucl Med Mol Imaging* **2022**, *49*, 1052–1062, doi:10.1007/s00259-021-05539-1.	Original study not in the field of interest
Peters, S.M.B.; Hofferber, R.; Privé, B.M.; de Bakker, M.; Gotthardt, M.; Janssen, M.; de Lange, F.; Muselaers, C.H.J.; Mehra, N.; Witjes, J.A.; et al. [68Ga]Ga-PSMA-11 PET Imaging as a Predictor for Absorbed Doses in Organs at Risk and Small Lesions in [177Lu]Lu-PSMA-617 Treatment. *Eur J Nucl Med Mol Imaging* **2022**, *49*, 1101–1112, doi:10.1007/s00259-021-05538-2.	Original study not in the field of interest
Dang, S.; Pereira, M.; Singh, N.; Shivdasani, D.; Roy, D.; Kesariya, J.; Rungta, R. An Unusual Case of Bilateral Ureteric Metastasis on PSMA PET-CT Scan in Carcinoma Prostate. *Clin Nucl Med* **2022**, *47*, e203–e204, doi:10.1097/RLU.0000000000003910.	Case report not in the field of interest
Aksu, A.; Çapa Kaya, G. Is SUV Corrected for Lean Body Mass Superior to SUV of Body Weight in 68Ga-PSMA PET/CT? *Mol Imaging Radionucl Ther* **2021**, *30*, 144–149, doi:10.4274/mirt.galenos.2021.59254.	Original study not in the field of interest
Mirzaei, S.; Lipp, R.; Zandieh, S.; Leisser, A. Single-Center Comparison of [64Cu]-DOTAGA-PSMA and [18F]-PSMA PET-CT for Imaging Prostate Cancer. *Curr Oncol* **2021**, *28*, 4167–4173, doi:10.3390/curroncol28050353.	Original study not in the field of interest
Leder, T.; Drescher, R.; Gühne, F.; Theis, B.; Freesmeyer, M. De Quervain Subacute Thyroiditis With Moderate PSMA Uptake Mimicking Thyroid Metastasis of Renal Cell Carcinoma. *Clin Nucl Med* **2022**, *47*, 221–222, doi:10.1097/RLU.0000000000003952.	Case report not in the field of interest

Machulkin, A.E.; Uspenskaya, A.A.; Zyk, N.U.; Nimenko, E.A.; Ber, A.P.; Petrov, S.A.; Polshakov, V.I.; Shafikov, R.R.; Skvortsov, D.A.; Plotnikova, E.A.; et al. Synthesis, Characterization, and Preclinical Evaluation of a Small-Molecule Prostate-Specific Membrane Antigen-Targeted Monomethyl Auristatin E Conjugate. *J Med Chem* **2021**, *64*, 17123–17145, doi:10.1021/acs.jmedchem.1c01157.	Original study not in the field of interest
Jiao, Y.; Xu, P.; Luan, S.; Wang, X.; Gao, Y.; Zhao, C.; Fu, P. Molecular Imaging and Treatment of PSMA-Positive Prostate Cancer with 99mTc Radiolabeled Aptamer-SiRNA Chimeras. *Nucl Med Biol* **2022**, *104*–105, 28–37, doi:10.1016/j.nucmedbio.2021.11.003.	Original study not in the field of interest
Filippi, L.; Braat, A.J. Theragnostics in Primary and Secondary Liver Tumors: The Need for a Personalized Approach. *Q J Nucl Med Mol Imaging* **2021**, *65*, 353–370, doi:10.23736/S1824-4785.21.03407-5.	Review not in the field of interest
Klein Nulent, T.J.W.; van Es, R.J.J.; Willems, S.M.; Braat, A.J.A.T.; Devriese, L.A.; de Bree, R.; de Keizer, B. First Experiences with 177Lu-PSMA-617 Therapy for Recurrent or Metastatic Salivary Gland Cancer. *EJNMMI Res* **2021**, *11*, 126, doi:10.1186/s13550-021-00866-8.	Original study not in the field of interest
Zhang, T.; Cai, J.; Xu, M.; Ma, X.; Wang, H.; Wang, M.; Han, Z.; Wang, J.; Smith, E.; Li, Z.; et al. Development of 18F-Labeled Vinyl Sulfone-PSMAi Conjugates as New PET Agents for Prostate Cancer Imaging. *Mol Pharm* **2022**, *19*, 720–727, doi:10.1021/acs.molpharmaceut.1c00743.	Original study not in the field of interest
Lu, X.; Wu, M.; Wang, S.; Hai, W.; Li, P. Development and Preliminary Evaluation of an Integrin A2β1-Targeted PET Probe as a Supplement and Alternative of PSMA Imaging for Prostate Cancer. *Bioorg Med Chem* **2022**, *54*, 116583, doi:10.1016/j.bmc.2021.116583.	Original study not in the field of interest
Dittrich, R.P.; De Jesus, O. Gallium Scan. In StatPearls; StatPearls Publishing: Treasure Island (FL), **2022**.	Book not in the field of interest
Klose, J.M.; Wosniack, J.; Iking, J.; Staniszewska, M.; Zarrad, F.; Trajkovic-Arsic, M.; Herrmann, K.; Fragoso Costa, P.; Lueckerath, K.; Fendler, W.P. Administration Routes for SSTR- / PSMA- and FAP-Directed Theranostic Radioligands in Mice. *J Nucl Med* **2022**, *jnumed*.121.263453, doi:10.2967/jnumed.121.263453.	Original study not in the field of interest
Kang, C.; Jiang, J.Y.; Lee, M.E.; Shen, L.; Mansberg, R. Incidental Intrahepatic Hepatocellular Cholangiocarcinoma Detected on 68Ga-PSMA PET/CT. *Clin Nucl Med* **2022**, *47*, e291–e293, doi:10.1097/RLU.0000000000003992.	Case report not in the field of interest
Kunikowska, J.; Czepczyński, R.; Pawlak, D.; Koziara, H.; Pełka, K.; Królicki, L. Expression of Glutamate Carboxypeptidase II in the Glial Tumor Recurrence Evaluated in Vivo Using Radionuclide Imaging. *Sci Rep* **2022**, *12*, 652, doi:10.1038/s41598-021-04613-w.	Original study not in the field of interest

Fedrigo, R.; Kadrmas, D.J.; Edem, P.E.; Fougner, L.; Klyuzhin, I.S.; Petric, M.P.; Bénard, F.; Rahmim, A.; Uribe, C. Quantitative Evaluation of PSMA PET Imaging Using a Realistic Anthropomorphic Phantom and Shell-Less Radioactive Epoxy Lesions. *EJNMMI Phys* **2022**, *9*, 2, doi:10.1186/s40658-021-00429-9.	Original study not in the field of interest
Nautiyal, A.; Jha, A.K.; Mithun, S.; Rangarajan, V. Dosimetry in Lu-177-PSMA-617 Prostate-Specific Membrane Antigen Targeted Radioligand Therapy: A Systematic Review. *Nucl Med Commun* **2022**, *43*, 369–377, doi:10.1097/MNM.0000000000001535.	Review not in the field of interest
Hasenauer, N.; Higuchi, T.; Deschler-Baier, B.; Hartrampf, P.E.; Pomper, M.G.; Rowe, S.P.; Fassnacht, M.; Buck, A.K.; Werner, R.A. Visualization of Tumor Heterogeneity in Advanced Medullary Thyroid Carcinoma by Dual-Tracer Molecular Imaging: Revealing the Theranostic Potential of SSTR- and PSMA-Directed Endoradiotherapy. *Clin Nucl Med* **2022**, *47*, 651–652, doi:10.1097/RLU.0000000000004082.	Case report not in the field of interest
Gurioli, G.; Conteduca, V.; Brighi, N.; Scarpi, E.; Basso, U.; Fornarini, G.; Mosca, A.; Nicodemo, M.; Banna, G.L.; Lolli, C.; et al. Circulating Tumor Cell Gene Expression and Plasma AR Gene Copy Number as Biomarkers for Castration-Resistant Prostate Cancer Patients Treated with Cabazitaxel. *BMC Med* **2022**, *20*, 48, doi:10.1186/s12916-022-02244-0.	Original study not in the field of interest
Knorr, K.; Oh, S.W.; Krönke, M.; Wurzer, A.; D'Alessandria, C.; Herz, M.; Weber, W.; Wester, H.-J.; Eiber, M.; Yusufi, N.; et al. Preclinical Biodistribution and Dosimetry and Human Biodistribution Comparing 18F-RhPSMA-7 and Single Isomer 18F-RhPSMA-7.3. *EJNMMI Res* **2022**, *12*, 8, doi:10.1186/s13550-021-00872-w.	Original study not in the field of interest
Wu, W.Y.; Yu, F.; Zhang, P.J.; Bu, T.; Fu, J.J.; Ai, S.Y.; You, Q.Q.; Shi, L.; Shao, G.Q.; Wang, F.; et al. 68Ga-DOTA-NT-20.3 Neurotensin Receptor 1 Positron Emission Tomography Imaging as a Surrogate for Neuroendocrine Differentiation of Prostate Cancer. *J Nucl Med* **2022**, jnumed.121.263132, doi:10.2967/jnumed.121.263132.	Original study not in the field of interest
Morawitz, J.; Kirchner, J.; Hertelendy, J.; Loberg, C.; Schimmöller, L.; Dabir, M.; Häberle, L.; Mamlins, E.; Antke, C.; Arsov, C.; et al. Is There a Diagnostic Benefit of Late-Phase Abdomino-Pelvic PET/CT after Urination as Part of Whole-Body 68 Ga-PSMA-11 PET/CT for Restaging Patients with Biochemical Recurrence of Prostate Cancer after Radical Prostatectomy? *EJNMMI Res* **2022**, *12*, 12, doi:10.1186/s13550-022-00885-z.	Original study not in the field of interest
Rosar, F.; Neher, R.; Burgard, C.; Linxweiler, J.; Schreckenberger, M.; Hoffmann, M.A.; Bartholomä, M.; Khreish, F.; Ezziddin, S. Upregulation of PSMA Expression by Enzalutamide in Patients with Advanced MCRPC. *Cancers (Basel)* **2022**, *14*, 1696, doi:10.3390/cancers14071696.	Original study not in the field of interest

Huang, Y.-T.; Tseng, N.-C.; Chen, Y.-K.; Huang, K.-H.; Lin, H.-Y.; Huang, Y.-Y.; Hwang, T.I.S.; Ou, Y.-C. The Detection Performance of 18 F-Prostate-Specific Membrane Antigen-1007 PET/CT in Primary Prostate Cancer : A Systemic Review and Meta-Analysis. *Clin Nucl Med* **2022**, *47*, 755–762, doi:10.1097/RLU.0000000000004228.	Review not in the field of interest
Luna-Gutiérrez, M.; Ocampo-García, B.; Jiménez-Mancilla, N.; Ancira-Cortez, A.; Trujillo-Benítez, D.; Hernández-Jiménez, T.; Ramírez-Nava, G.; Hernández-Ramírez, R.; Santos-Cuevas, C.; Ferro-Flores, G. Targeted Endoradiotherapy with Lu2O3-IPSMA/-IFAP Nanoparticles Activated by Neutron Irradiation: Preclinical Evaluation and First Patient Image. *Pharmaceutics* **2022**, *14*, 720, doi:10.3390/pharmaceutics14040720.	Original study not in the field of interest
Gaudreault, M.; Chang, D.; Hardcastle, N.; Jackson, P.; Kron, T.; Hanna, G.G.; Hofman, M.S.; Siva, S. Utility of Biology-Guided Radiotherapy to De Novo Metastases Diagnosed During Staging of High-Risk Biopsy-Proven Prostate Cancer. *Front Oncol* **2022**, *12*, 854589, doi:10.3389/fonc.2022.854589.	Original study not in the field of interest
Abubakar, S.; Al Riyami, K.; Jain, A.; Jayakrishnan, V.; Tag, N. Reverse Liver Spleen Uptake on [68Ga]Ga-PSMA-11 PET/CT. *Clin Nucl Med* **2022**, doi:10.1097/RLU.0000000000004285.	Case report not in the field of interest
Basuli, F.; Phelps, T.E.; Zhang, X.; Woodroofe, C.C.; Roy, J.; Choyke, P.L.; Swenson, R.E.; Jagoda, E.M. Fluorine-18 Labeled Urea-Based Ligands Targeting Prostate-Specific Membrane Antigen (PSMA) with Increased Tumor and Decreased Renal Uptake. *Pharmaceuticals (Basel)* **2022**, *15*, 597, doi:10.3390/ph15050597.	Original study not in the field of interest
Lu, Q.; Long, Y.; Fan, K.; Shen, Z.; Gai, Y.; Liu, Q.; Jiang, D.; Cai, W.; Wan, C.; Lan, X. PET Imaging of Hepatocellular Carcinoma by Targeting Tumor-Associated Endothelium Using [68Ga]Ga-PSMA-617. *Eur J Nucl Med Mol Imaging* **2022**, doi:10.1007/s00259-022-05884-9.	Original study not in the field of interest
Ayala Soriano, C.; Benitez Barzaga, M.; Chhina, A.; Jain, M.; Nava, V.E. Hepatoid Prostatic Carcinoma with Adrenal Metastasis and Novel Genetic Alterations. *Diagn Cytopathol* **2022**, doi:10.1002/dc.25006.	Case report not in the field of interest
Xue, S.; Gafita, A.; Dong, C.; Zhao, Y.; Tetteh, G.; Menze, B.H.; Ziegler, S.; Weber, W.; Afshar-Oromieh, A.; Rominger, A.; et al. Application of Machine Learning to Pretherapeutically Estimate Dosimetry in Men with Advanced Prostate Cancer Treated with 177Lu-PSMA I&T Therapy. *Eur J Nucl Med Mol Imaging* **2022**, doi:10.1007/s00259-022-05883-w.	Original study not in the field of interest

Wrenger, R.; Jüptner, M.; Marx, M.; Zhao, Y.; Zuhayra, M.; Caliebe, A.; Osmonov, D.; Lützen, U. Pre- and Intratherapeutic Predictors of Overall Survival in Patients with Advanced Metastasized Castration-Resistant Prostate Cancer Receiving Lu-177-PSMA-617 Radioligand Therapy. *BMC Urol* **2022**, *22*, 96, doi:10.1186/s12894-022-01050-3.	Original study not in the field of interest
Lu, Y.; Li, C. Incidental Findings of Coexisting Metastatic Pancreatic Cancer in Patients With Prostate Cancer on 18F-Prostate-Specific Membrane Antigen PET/CT. *Clin Nucl Med* **2022**, doi:10.1097/RLU.0000000000004338.	Case report not in the field of interest
Sergeeva, O.; Zhang, Y.; Julian, W.; Sasikumar, A.; Awadallah, A.; Kenyon, J.; Shi, W.; Sergeev, M.; Huang, S.; Sexton, S.; et al. Imaging of Tumor-Associated Vascular Prostate-Specific Membrane Antigen in Woodchuck Model of Hepatocellular Carcinoma. *Gastro Hep Adv* **2022**, *1*, 631–639, doi:10.1016/j.gastha.2022.04.014.	Original study not in the field of interest
Christ, S.M.; Pohl, K.; Muehlematter, U.J.; Heesen, P.; Kühnis, A.; Willmann, J.; Ahmadsei, M.; Badra, E.V.; Kroeze, S.G.C.; Mayinger, M.; et al. Imaging-Based Prevalence of Oligometastatic Disease: A Single-Center Cross-Sectional Study. *Int J Radiat Oncol Biol Phys* **2022**, S0360-3016(22)00719-2, doi:10.1016/j.ijrobp.2022.06.100.	Original study not in the field of interest
Rosar, F.; Schaefer-Schuler, A.; Bartholomä, M.; Maus, S.; Petto, S.; Burgard, C.; Privé, B.M.; Franssen, G.M.; Derks, Y.H.W.; Nagarajah, J.; et al. [89Zr]Zr-PSMA-617 PET/CT in Biochemical Recurrence of Prostate Cancer: First Clinical Experience from a Pilot Study Including Biodistribution and Dose Estimates. *Eur J Nucl Med Mol Imaging* **2022**, doi:10.1007/s00259-022-05925-3.	Original study not in the field of interest
Filippi, L.; Braat, A.J.; Schillaci, O. The Era of Prostate-Specific Membrane Antigen (PSMA)-Based Theranostics for Hepatocellular Carcinoma Is Upcoming: Are We Ready for It? *Eur J Nucl Med Mol Imaging* **2022**, doi:10.1007/s00259-022-05928-0.	Editorial in the field of interest
Sharma, P.; Watts, A.; Singh, H. Comparison of Internal Dosimetry of 18F-PSMA-1007 and 68Ga-PSMA-11-HBED-CC. *Clin Nucl Med* **2022**, doi:10.1097/RLU.0000000000004353.	Original study not in the field of interest
Zhang, X.; Sun, S.; Miao, Y.; Yuan, Y.; Zhao, W.; Li, H.; Wei, X.; Huang, C.; Hu, X.; Wang, B.; et al. Docetaxel Enhances the Therapeutic Efficacy of PSMA-Specific CAR-T Cells against Prostate Cancer Models by Suppressing MDSCs. *J Cancer Res Clin Oncol* **2022**, doi:10.1007/s00432-022-04248-y.	Original study not in the field of interest
Wang, G.; Zhou, M.; Zang, J.; Jiang, Y.; Chen, X.; Zhu, Z.; Chen, X. A Pilot Study of 68 Ga-PSMA-617 PET/CT Imaging and 177Lu-EB-PSMA-617 Radioligand Therapy in Patients with Adenoid Cystic Carcinoma. *EJNMMI Res* **2022**, *12*, 52, doi:10.1186/s13550-022-00922-x.	Original study not in the field of interest
